# Plant *N*-acylethanolamines play a crucial role in defense and its variation in response to elevated CO_2_ and temperature in tomato

**DOI:** 10.1093/hr/uhac242

**Published:** 2022-10-26

**Authors:** Zhangjian Hu, Junying Shi, Shuxian Feng, Xiaodan Wu, Shujun Shao, Kai Shi

**Affiliations:** Hainan Institute, Zhejiang University, Yazhou Bay Science and Technology City, Sanya 572025, China; Department of Horticulture, Zhejiang University, 310058, China; Department of Horticulture, Zhejiang University, 310058, China; Department of Horticulture, Zhejiang University, 310058, China; Analysis Center of Agrobiology and Environmental Science, Zhejiang University, Hangzhou, 310058, China; Department of Horticulture, Zhejiang University, 310058, China; Hainan Institute, Zhejiang University, Yazhou Bay Science and Technology City, Sanya 572025, China; Department of Horticulture, Zhejiang University, 310058, China

## Abstract

The ubiquitous lipid-derived molecules *N-*acylethanolamines (NAEs) have multiple immune functions in mammals, but their roles and mechanisms in plant defense response during changing environment remain largely unclear. Here, we found that exogenous NAE18:0 and NAE18:2 promoted defense against the necrotrophic pathogen *Botrytis cinerea* but suppressed defense to the hemi-biotrophic pathogen *Pseudomonas syringae* pv. *tomato* (*Pst*) DC3000 in tomato. The knocking-down and overexpression function analysis of the pathogen-responsive NAE synthetic gene *PHOSPHOLIPASE Dγ* (*PLDγ*) and hydrolytic gene *FATTY ACID AMID HYDROLASE 1* (*FAAH1*) revealed that the NAE pathway is crucial for plant defense response. Using exogenous applications and SA-abolished NahG plants, we unveiled the antagonistic relationship between NAE and SA in plant defense response. Elevated CO_2_ and temperature significantly changed the NAE pathway in response to pathogens, while inhibition of the NAE pathway led to the alternation of environment-mediated defense variations against *Pst* DC3000 in tomato, indicating that NAE pathway is associated with plant defense variations in response to elevated CO_2_ and temperature. The results herein reveal a new function of NAE in plant defense, and its involvement in environment-mediated defense variation in tomato. These findings shed light on the NAE-based plant defense, which may have relevance to crop disease management in future changing climate.

## Introduction

*N*-Acylethanolamines (NAEs) belong to a type of fatty acid amides, having an ethanolamine head group linked to an acyl group through an amide bond [1]. As lipid-derived signaling molecules, NAEs appear to be ubiquitous in plants, invertebrates, and mammals. In mammals, several types of NAEs, such as endocannabinoids, have been demonstrated to modulate a variety of physiological processes, especially innate immunity via G protein-coupled receptors [2]. In contrast, the knowledge of the physiological functions of NAEs in plants is fragmentary. The concentrations of endogenous NAEs have been found higher in seeds than seedlings, and the accumulation and metabolism of NAEs are primarily associated with seed germination, seedling development, and chlorophyll biosynthesis [1, 3]. However, whether the NAEs are involved in plant defense against pathogenic microbes remains largely unclear.

In plants, NAEs show high diversity in acyl chain length and the degree of saturation depending on the tissue types, development stages, and pathological conditions [4]. Different molecular types of NAEs are distinguished by the length of the *N*-linked acyl chain (C12 to C18) and the unsaturation degree of these chains (from 0 to 3) [5]. Specific NAE is abbreviated in terms of the length of carbons and the number of double bonds in the acyl chain. Long-chain NAEs (C16 to C18) are predominant types of NAE in seeds, among which C18 NAEs are the most abundant NAE in several plant species, including *Arabidopsis thaliana*, soybean, and tomato [3, 6].

NAEs are biosynthesized via a one-step reaction from *N*-acylphosphatidylethanolamines (NAPEs) in plants, and it is known that NAPEs are catalyzed by a Ca^2+^-activated NAPE-hydrolyzing phospholipase D (NAPE-PLD) in animals [3]. *In vitro* evidence suggests that NAPEs are most likely catalyzed by subgroups of PLD β/γ in plants, thereby generating phosphatidic acid (PA) and NAEs [1]. Arabidopsis contains 12 PLD homologs that are clustered into α to ζ subgroups according to conserved domain structures [7], but the identification of PLDs and their association with NAE biosynthesis remain largely unclear in other plant species. Limited studies showed that the role of PLD in plant defense is mainly associated with PLDβ. For example, rice *OsPLDβ1*-knockdown plants show enhanced resistance to several major rice pathogens, such as the blast fungus *Pyricularia grisea* and the bacterial blight *Xanthomonas oryzae* pv. *oryzae* [8]. Similarly, silencing *AtPLDβ1* leads to enhanced plant defense against both virulent and avirulent *Pseudomonas syringae* pv. *tomato* (*Pst*) DC3000 strains in Arabidopsis [9]. In contrast, the *Atpldβ1* mutants are more susceptible to necrotrophic fungus *Botrytis cinerea* [9]. Several other subfamilies of PLD, including *PLDα*, *PLDγ*, and *PLDδ* are also responsive to various plant pathogens at the transcript levels [10, 11], but it is still not clear whether these PLDs are involved in plant defense, especially against different type of pathogens.

Fatty acid amide hydrolase (FAAH) hydrolyzes NAEs to the production of ethanolamine and its corresponding free fatty acids (FFAs), which terminates their actions [12]. FAAH is the unique integral membrane protein in the amidase signature superfamily, and appears to have efficient activity towards a wide range of NAEs that includes unsubstituted forms and oxygenated derivatives [13, 14]. Except for NAEs, the microbe-derived quorum-sensing molecules *N*-acyl homoserine lactones (AHLs) also appear to serve as the prospective hydrolysis substrates for FAAHs [15, 16]. FAAHs in angiosperms are divided into two groups according to the structural divergence in conserved substitutions in amino acid residues surrounding the substrate-binding pocket and the cytosolic access channel [13]. Like animal FAAHs, plant FAAHs are capable of hydrolyzing NAEs *in vitro*, and Arabidopsis *faah* knockout mutants show increased endogenous NAE accumulation *in vivo* [17, 18]. So far, the majority of FAAH functions in plants are based on the studies of Arabidopsis *FAAH* overexpression lines that display enhanced seedling growth, increased cell/organ size, and early flowering [18, 19]. In the plant-pathogen interaction study, *AtFAAH* overexpression lines show compromised plant defense against *P. syringea*, revealing that FAAH is essential for plant defense response [20]. However, *AtFAAH* knockout mutants exhibit similar disease susceptibility to wild type plants [20]. Thus, it remains unclear whether FAAH functions as an integral part of NAE metabolism in defense response, particularly in other plant species.

Plants have evolved sophisticated mechanisms to protect themselves against pathogens by eliciting Ca^2+^ flux, reactive oxygen species (ROS) burst, lipid-derived signaling, plant hormone accumulations, etc. [21]. Of these, the salicylic acid (SA) defense pathway is a key component in the regulation of plant resistance to various pathogenic microbes, especially (hemi-)biotrophic pathogens [22]. Previous studies have implicated both PLD- and FAAH-associated SA signaling as critical components in plant defense response; nonetheless, the link between lipid-derived NAE and SA singling remains elusive [9, 20]. In Arabidopsis, the *AtPLDβ1* knockout mutants exhibit a high SA level in response to *Pst* DC3000 infection [9]. On the contrary, *AtFAAH* overexpression lines show a lower content of SA no matter with or without *Pst* DC3000 attack [20]. As the NAE synthase and hydrolase, PLD and FAAH are thought to play the opposite roles in NAE metabolism, respectively. However, both NAE-related enzymes show a similar effect on SA-mediated defense signaling, signifying the complexity of their functions. Therefore, it is worth exploring whether and how NAE and SA signaling crosstalk in plant defense response.

Tomato is a major cultivated vegetable crop worldwide and is also regarded as a model organism in plant biology [23, 24]. During cultivation and post-harvest management, tomato is threatened by over hundreds of pests and diseases. In particular, hemibiotrophic bacterial pathogen *Pst* DC3000 and necrotrophic fungal pathogen *B. cinerea*, which cause foliar speck disease and grey mold disease, respectively, have special economic importance [25]. In the climate change era, anthropogenic emissions and global warming are generating a rapid increase in CO_2_ and air temperatures that will have a profound impact on agriculture and natural ecosystems. However, it remains elusive how plants operate defense variations to changing environments. Here, we demonstrated that NAE18:2 promoted plant defense against *B. cinerea* but they suppressed resistance to *Pst* DC3000. This finding was also supported by knocking-down and overexpression function analyses of NAE synthase encoding gene *PLDγ* and hydrolase encoding gene *FAAH1*. Our results also suggest that NAE and its metabolism pathway are associated with SA-mediated defense signaling, and are involved in plant defense variations in response to elevated temperature and CO_2_. These findings shed light on NAE-based plant defense response, which could put forward new strategies for increasing crop resistance in the era of changing climate.

## Results

### Exogenous NAEs promote plant defense against *B. cinerea* but attenuate immune response against *Pst* DC3000

**Figure 1:**
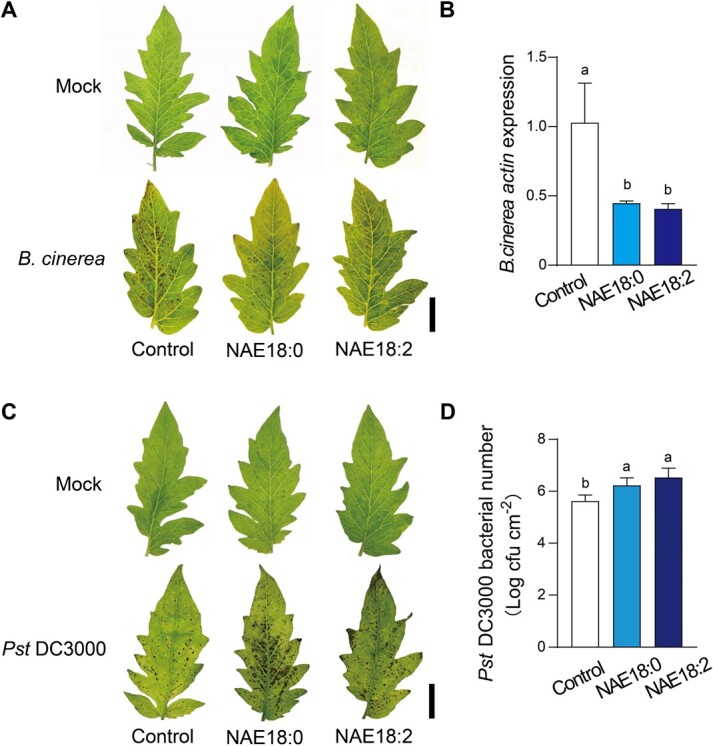
NAEs differentially modulate plant defense to *B. cinerea* and *Pst* DC3000. **A** Representative leaf images for disease symptoms at 3 days post *B. cinerea* inoculation (dpi). Bar, 2 cm. **B** Relative *B. cinerea actin* transcript abundance in tomato leaves at 1 dpi. **C** Representative leaf images for disease symptoms at 3 dpi. Bar, 2 cm. **D** Bacterial growth of *Pst* DC3000 in tomato leaves at 3 dpi. Four-week-old tomato plants were sprayed by 80 μM NAE18:0, NAE18:2 or corresponding ethanol solution control once per d. After 2 d of chemical treatments, the plants were subjected to *Pst* DC3000, *B.cinerea*, or corresponding mock inoculations. The data are presented in **B** and **D** as mean values ± standard deviation (SD); *n* = 3 in **B**, 5 in **D**. Different letters indicate significant differences between treatments (*P* < 0.05, Tukey’s test).

The divergence in pathogen lifestyle and the complexity of plant-pathogen interactions eventually shape the defense response of plants. To characterize the functions of NAEs in plant defense against different type of pathogens, tomato plants were sprayed with NAE18:0 or NAE18:2, and 2 days later they were subjected to pathogen inoculation. Based on the disease symptoms at 3 days post-inoculation (dpi), plants pretreated with NAE18:0 or NAE18:2 displayed enhanced disease resistance against *B. cinerea* (Fig. 1A). Consistent with the disease phenotype on leaves, less transcript abundance of *B. cinerea Actin* were quantified in both NAE18:0- and NAE18:2-pretreated leaves (Fig. 1B). However, both NAE18:0 and NAE18:2 induced more severe disease symptoms in tomato leaves when challenged with *Pst* DC3000 inoculation (Fig. 1C). A higher *Pst* DC3000 bacterial population was counted in both NAE18:0- and NAE18:2-pretreated leaves (Fig. 1D). Together, these data indicated that NAEs differentially modulated plant defense to *B. cinerea* and *Pst* DC3000 attacks.

### Phylogenetic analysis of NAE pathway-related genes and changes in their transcript abundances in response to pathogen inoculation

**Figure 2:**
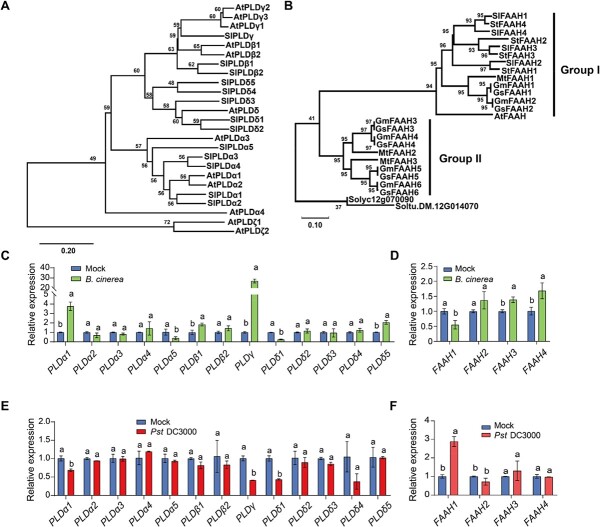
Transcript changes of tomato *PLD* and *FAAH* genes in response to *Pst* DC3000 and *B. cinerea* inoculation. **A** Phylogenetic tree analysis of phospholipase D (PLD) family from *Solanum lycopersicum* (Sl) and *Arabidopsis thaliana* (At). **B** Phylogenetic tree analysis of fatty acid amid hydrolase family from *S. lycopersicum* (Sl), *S. tuberosum* (St), *Glycine max* (Gm), *Glycine soja* (Gs), *Medicago truncatula* (Mt), and *A. thaliana* (At). Solyc12g070090 from *S. lycopersicum* and Soltu.DM. 12G014070 from *S. tuberosum* served as the closest amidase protein superfamily members. Amino acid sequence alignments and phylogenetic tree constructions were performed with the MEGA-X program via a consensus neighbor-joining tree approach. Bootstrap values calculated from 1000 trials are shown at each node. The extent of divergence according to the scale (relative units) is indicated at the bottom. **C** Transcript abundance of tomato *PLD* genes in response to *B. cinerea* inoculation. **D** Transcript abundance of tomato *FAAH* genes in response to *B. cinerea* inoculation. **E** Transcript abundance of tomato *PLD* genes in response to *Pst* DC3000 inoculation. **F** Transcript abundance of tomato *FAAH* genes in response to *Pst* DC3000 inoculation. Four-week-old tomato plants were subjected to *Pst* DC3000, *B.cinerea*, or corresponding mock spray inoculation. Leaf samples were collected at 12 hours post-inoculation for qRT-PCR analysis. The transcript abundance of each gene under mock-inoculated condition was defined as 1. The data are presented in **C**–**F** as mean values ± standard deviation (SD); *n* = 3. Different letters indicate significant differences between treatments (*P* < 0.05, Tukey’s test).

According to the studies in Arabidopsis, there are 12 PLD members classified into α-ζ types based on predicted amino acid sequences, and one unique FAAH member that has the capability of hydrolyzing NAEs [7, 26]. In the tomato genome, 13 *PLD* genes were identified based on homology to Arabidopsis predicted amino acid sequences of PLDs (Fig. 2A), In Arabidopsis, AtPLDβ/γ are potentially involved in NAE formation, and we found two β-type PLDs (PLDβ1 and PLDβ2) and a unique PLDγ in tomato (Fig. 2A). Meanwhile, 4 *FAAH* genes were discovered in tomato genome based on the cluster analysis with the reported amino acid sequences of FAAH homologs in *Solanum tuberosum*, *Glycine max*, *Glycine soja*, *Medicago truncatula*, and *Arabidopsis. thaliana* (Fig. 2B). All these tomato FAAHs belong to group I FAAH (Fig. S1, see online supplementary material), and hereafter are referred to as tomato FAAH1–4.

To investigate whether *PLD*s and *FAAH*s were responsive to pathogen infections, transcript abundance of these gene families was analysed. The gene expression levels of several *PLD*s, such as *PLDα1*, *PLDβ1*, *PLDγ*, and *PLDδ5*, were significantly induced by *B. cinerea* infection at 12 hours post-inoculation (hpi), among which *PLDγ* showed the highest (about 26-fold) induction (Fig. 2C). The transcript abundance of other *PLD* genes showed no remarkable changes, or even decreased (e.g. *PLDα5* and *PLDδ1*) in response to *B. cinerea* infection. In the case of the NAE-hydrolysis process, the transcript levels of one gene, *FAAH1,* were significantly suppressed, while that of other three *FAAH* genes were slightly induced by *B. cinerea* at 12 hpi (Fig. 2D). When tomato plants were subjected to *Pst* DC3000 inoculation, the gene expression levels of *PLDα1/γ/δ1* were significantly suppressed, while no significant changes were observed in other *PLD* genes (Fig. 2E). In response to *Pst* DC3000 inoculation, the transcript abundance of *FAAH1* remarkably increased at 12 hpi. Based on these results, several pathogen-responsive *PLD* and one *FAAH* genes, i.e. *PLDα1/γ/δ1* and *FAAH1*, were selected for further investigation to explore their roles in plant defense against different type of pathogens.

### Functions of *PLD* and *FAAH* genes in plant defense

**Figure 3:**
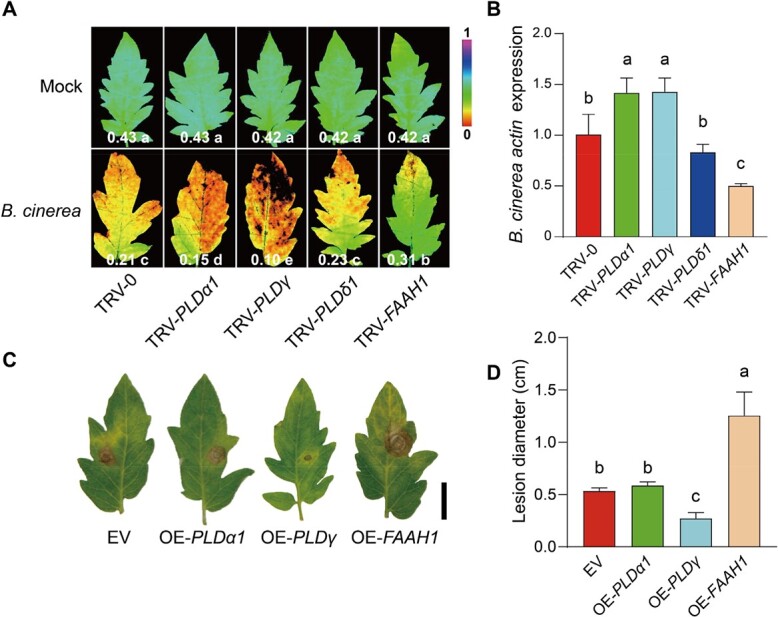
The different roles of PLDs and FAAH1 in plant defense against *B. cinerea.***A** Pseudo-color images showing Ф_PSII_ in indicated gene silencing tomato leaves at 3 days post *B. cinerea* inoculation (dpi). The color gradient scale at right indicates the magnitude of the fluorescence signal. The data at the bottom of each image presents the mean Ф_PSII_ values (*n* ≥ 6). **B** Relative *B. cinerea actin* transcript abundance in indicated gene silencing tomato leaves at 1 dpi. **C** Effect of transient overexpression of indicated genes on *B. cinerea* growth in tomato leaves. Bar, 2 cm. EV, empty vector. **D** Diameter quantification of *B. cinerea* infection lesions. The data are presented in **B** and **D** as mean values, and ± standard deviation (SD), *n* = 3 in **B**, 6 in **D**. Different letters indicate significant differences between treatments (*P* < 0.05, Tukey’s test).

We generated target gene silenced tomato plants via virus-induced gene silencing (VIGS) and transiently overexpressed the target genes in tomato to examine their roles in plant defense response. Silencing target gene didn’t affect the transcript abundance of their homologous genes (Fig. S2, see online supplementary material). Typically, infections with foliar phytopathogens cause irreversible damage to the host photosynthetic system, so the photochemical quantum yield at photosystem II (Ф_PSII_) was measured to assess the level of disease severity [25]. Firstly, we investigated the defense roles of pathogen-responsive genes in VIGS plants in response to necrotrophic *B. cinerea* infection. As shown in Fig. 3A, compared with control TRV-0 plants, the TRV-*PLDα1* and TRV-*PLDγ* plants exhibited lower Ф_PSII_ values, while TRV-*FAAH1* plants showed a slight decline in Ф_PSII_ at 3 dpi with *B. cinerea* inoculation. Consistent with Ф_PSII_ values, a higher transcript abundance of *B. cinerea actin* was detected in TRV-*PLDα1* and TRV-*PLDγ* plants, conversely a lower *B. cinerea actin* expression was found in TRV-*FAAH1* plants (Fig. 3B). We then transiently overexpressed each of these three genes driven by CaMV 35S promoter in tomato leaves followed by the inoculation with *B. cinerea.* When we overexpressed *PLDγ* in tomato leaves, *B. cinerea*-induced necrotic lesions were largely inhibited in the overexpression area, but *FAAH1* overexpression promoted the *B. cinerea*-induced necrotic lesions (Fig. 3C and D). Unlike the silencing approach, *PLDα1*-overexpressed leaves showed a smilar *B. cinerea* symptom to empty vector-inoculated control (Fig. 3C and D). These results indicated that PLDγ promoted, but FAAH1 suppressed the plant resistance to *B. cinerea* in tomato leaves.

**Figure 4:**
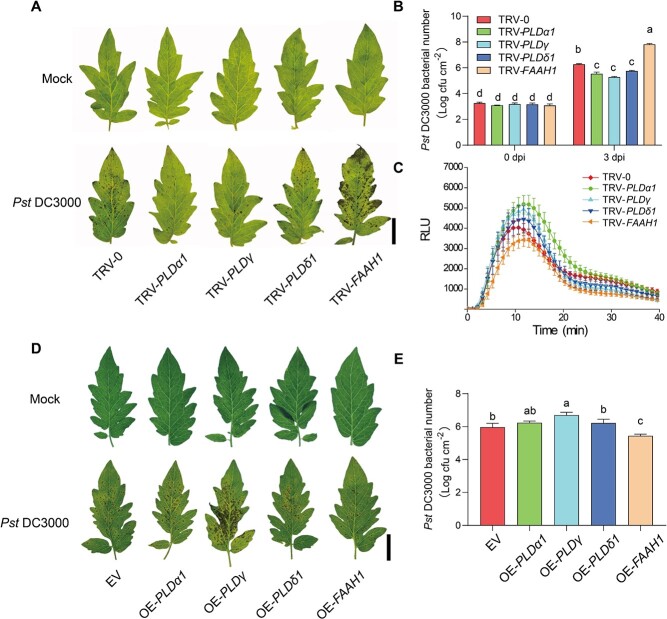
The different roles of PLDs and FAAH1 in plant defense against *Pst* DC3000. **A** Representative leaf images in tomato VIGS plants for disease symptoms at 3 days post *Pst* DC3000 inoculation (dpi). Bar, 2 cm. **B** Bacterial growth of *Pst* DC3000 in tomato leaves at 0 dpi and 3 dpi. **C** Induction of ROS in indicating plants after treatment with 100 nM flg22. **D** Representative images in tomato transient overexpression leaves for disease symptoms at 3 dpi. Bar, 2 cm. EV, empty vector. **E** Bacterial growth of *Pst* DC3000 in tomato leaves at 3 dpi. The data are presented in **B**, **C** and **E** as mean values, and ± standard deviation (SD); *n* = 5 in **B** and **E**, ≥12 in **C**. Different letters indicate significant differences between treatments (*P* < 0.05, Tukey’s test).

To investigate the potential roles of these genes in plant defense to *Pst* DC3000, the VIGS plants were subjected to *Pst* DC3000 inoculation. TRV-*PLDα1*, TRV-*PLDγ*, and TRV-*PLDδ1* plants exhibited a significant alleviation of disease symptoms as evidenced by the decreased bacterial growth, whereas TRV-*FAAH1* plants showed a higher susceptibility to *Pst* DC3000 than TRV-0 plants (Fig. 4A and B). Consistently, TRV-*PLDα1* and TRV-*PLDγ* plants exhibited a stronger ROS burst in response to flg22 treatment compared with TRV-0 plants, whereas the flg22-induced ROS burst was inhibited in TRV-*FAAH1* plants (Fig. 4C). By the transient overexpression in tomato leaves, we found that overexpression of *PLDγ* exacerbated the *Pst* DC3000-induced damage, whereas the *FAAH1* overexpression contributed to an opposite effect (Fig. 4D and E).

**Figure 5:**
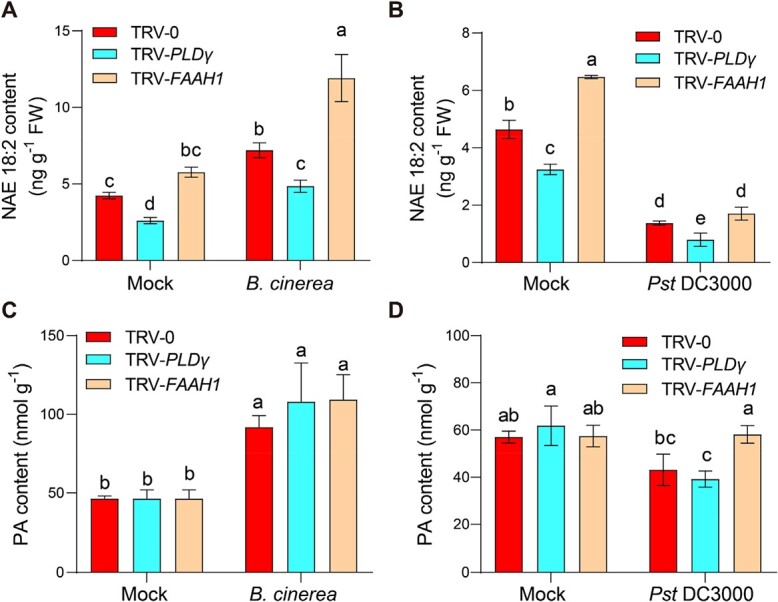
Effects of PLDγ and FAAH1 in NAE and PA contents during plant defense responses. (**A**) Effects of *B. cinerea* inoculation on endogenous NAE18:2 content in tomato plants. (**B**) Effects of *Pst* DC3000 inoculation on endogenous NAE18:2 content in tomato plants. (**C**) Effects of *B. cinerea* inoculation on PA content in tomato plants. (**D**) Effects of *Pst* DC3000 inoculation on PA content in tomato plants. Five–week–old indicated VIGS tomato plants were subjected to *Pst* DC3000, *B.cinerea*, or corresponding mock spray inoculation. Leaf samples were collected at 24 hpi for enzyme activity analysis. The data are presented as mean values ± standard deviation (SD); *n* = 3. Different letters indicate significant differences between treatments (*P* < 0.05, Tukey’s test).

To further investigate the relationship between the NAE accumulation and pathogen infection, the endogenous NAE18:2 contents were quantified in VIGS plants using LC–MS analyses. NAE18:2 contents decreased in TRV-*PLDγ* plants but slightly increased in TRV-*FAAH1* plants compared with those in TRV-0 control plants in the absence of pathogen infection (Fig. 5A). In response to *B. cinerea* attacks, NAE18:2 content significantly increased in TRV-*FAAH1* plants, and *PLDγ* silencing resulted in the suppression of *B. cinerea*-induced NAE18:2 accumulation (Fig. 5A). Meanwhile, *Pst* DC3000 inoculation significantly suppressed NAE 18:2 contents in TRV-0, TRV-*PLDγ*, and TRV-*FAAH1* plants, and there was no significant difference in NAE18:2 contents between TRV-0 and TRV-*FAAH1* plants after *Pst* DC3000 inoculation (Fig. 5B). As another hydrolysis product of NAPE, the PA contents were only increased by *B. cinerea* but not by *Pst* DC3000 inoculation, and showed a similar level in TRV-0 and TRV-*PLDγ* plants with or without pathogen attacks (Fig. 5C and D). It might be explained that PA biosynthesis was not fully dependent on NAPE hydrolysis.

### NAE pathway is linked to plant defense variations in response to elevated CO_2_ and temperature conditions

Lipid-derived signaling plays a key role in plant tolerance to temperature stress by affecting plant heat sensing, whereas high atmospheric CO_2_ concentrations can also trigger lipid signaling and modifications [27–29]. Thus, it was hypothesized that NAE signaling was potentially linked to defense variations in response to these environmental factors.

**Figure 6:**
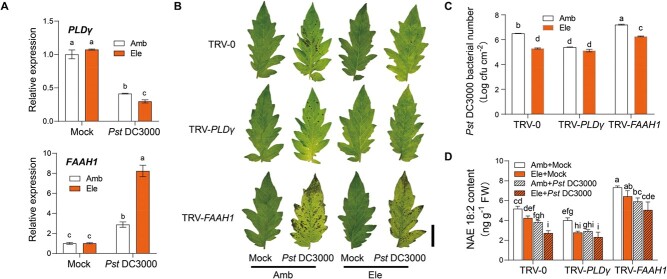
NAE pathway is involved in elevated CO_2_-mediated plant defense. **A** Transcript abundance of tomato *PLDγ* and *FAAH1* under ambient CO_2_ (Amb, 400 μmol mol^−1^) or elevated CO_2_ (Ele, 800 μmol mol^−1^) conditions at 12 hours post *Pst* DC3000 inoculation. **B** Representative leaf images for disease symptoms in VIGS tomato plants at 3 days post *Pst* DC3000 inoculation (dpi) under ambient CO_2_ or elevated CO_2_ conditions. Bar, 2 cm. **C** Bacterial growth of *Pst* DC3000 in tomato leaves at 3 dpi. **D** Changes in NAE18:2 in tomato plants. Five-week-old tomato plants were treated with different CO_2_ concentrations for 2 d before *Pst* DC3000 inoculation. Leaf samples were collected at 1 dpi with *Pst* DC3000 for NAE quantification. The data are presented as mean values ± standard deviation (SD); *n* = 3, except for 5 in **C**. Different letters indicate significant differences between treatments (*P* < 0.05, Tukey’s test).

To test the above hypothesis, we first analysed the transcript abundance of *PLDγ* and *FAAH1* when tomato plants were subjected to *Pst* DC3000 inoculation under different CO_2_ conditions. As shown in Fig. 6A, elevated CO_2_ enhanced the gene suppression of *PLDγ* in response to *Pst* DC3000 attack, but largely promoted *Pst* DC3000-induced *FAAH1* gene expression. Then, TRV-*PLDγ*, TRV-*FAAH1,* and TRV-0 control plants were subjected to ambient and elevated CO_2_ treatments for 2 days before *Pst* DC3000 inoculation (Fig. 6B and C). Similar to plant defense responses under ambient CO_2_ conditions, TRV-*PLDγ* plants still showed robust defense against bacterial infection but TRV-*FAAH1* plants showed at a high disease susceptibility level in elevated CO_2_. Interestingly, elevated CO_2_ failed to further increase plant defense in TRV-*PLDγ* plants. However, the disease symptoms in TRV-*FAAH1* were slightly but still significantly alleviated by elevated CO_2_ compared with that in TRV-0 control plants. Further analysis of NAE contents showed that elevated CO_2_ generally suppressed NAE18:2 contents in response to *Pst* DC3000 attacks, especially in TRV-0 plants (Fig. 6D). In addition, TRV- *PLDγ* plants maintained low NAE levels irrespective of the CO_2_ conditions (Fig. 6D). Hence, these results implied that elevated CO_2_-induced plant defense was linked to the NAE metabolism pathway.

**Figure 7:**
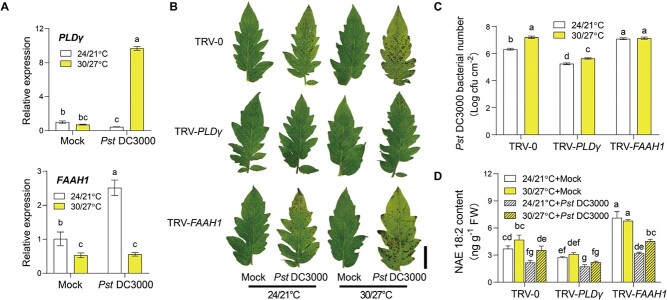
NAE pathway is essential for elevated temperature-mediated plant defense. **A** Transcript abundance of tomato *PLDγ* and *FAAH1* under ambient temperature (24/21°C, day/night) or elevated temperature (30/27°C, day/night) conditions at 12 hours post *Pst* DC3000 inoculation. **B** Representative leaf images for disease symptoms in VIGS tomato plants at 3 days post *Pst* DC3000 inoculation (dpi) under ambient temperature or elevated temperature conditions. Bar, 2 cm. **C** Bacterial growth of *Pst* DC3000 in tomato leaves at 3 dpi. **D** Changes in NAE18:2 in tomato plants. Five-week-old tomato plants were treated with different temperatures for 2 d before *Pst* DC3000 inoculation. Leaf samples were collected at 1 dpi with *Pst* DC3000 for NAE quantification. The data are presented as mean values ± standard deviation (SD); *n* = 3, except for 5 in **C**. Different letters indicate significant differences between treatments (*P* < 0.05, Tukey’s test).

Whether the plant defense variation caused by elevated temperatures was linked to the alterations in the NAE metabolism pathway was also investigated. In *Pst* DC3000-inoculated tomato plants, the gene expression of *PLDγ* was significantly promoted by the elevated temperature at 12 hpi, whereas the effect was the opposite in the case of *FAAH1* gene expression (Fig. 7A). To test whether the NAE pathway had a biological effect on temperature-mediated plant defense variations, VIGS plants were cultured in different temperatures for 2 d and then further subjected to pathogen inoculation (Fig. 7B and C). Elevated temperatures largely induced susceptibility to *Pst* DC3000 in tomato plants, which was consistent with a previous study [30]. This phenomenon was also found in TRV-*PLDγ* plants despite the induction of disease susceptibility was slight but significant. Interestingly, the disease susceptibility of TRV-*FAAH1* plants was similar to control TRV-0 plants at elevated temperatures, indicating that the susceptibility of TRV-*FAAH1* plants was not further aggravated by elevated temperature. Moreover, NAE content analysis revealed that elevated temperature promoted NAE18:2 accumulations in TRV-0 and TRV-*PLDγ* plants (Fig. 7D). Overall, these results suggested that elevated temperature modulated the NAE pathway and further led to attenuated plant defense against bacterial pathogens.

### NAE plays an antagonistic role with SA in plant defense

**Figure 8:**
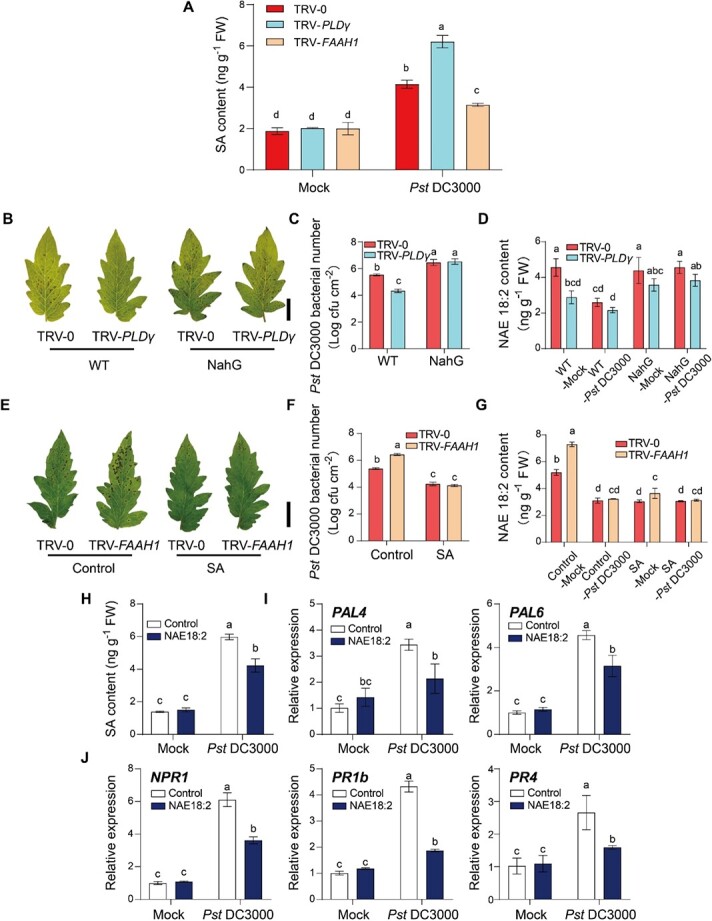
NAE pathway is associated with SA-mediated defense signaling. **A** Changes of endogenous SA in VIGS plants at 24 hours post *Pst* DC3000 inoculation (hpi). **B**–**D** Effects of *PLDγ* silencing on plant defense in NahG and its control plants against *Pst* DC3000. **B** Representative leaf images for disease symptoms at 3 days post *Pst* DC3000 inoculation (dpi). Bar, 2 cm. **C** Bacterial growth of *Pst* DC3000 in tomato leaves at 3 dpi. **D** Changes of NAE18:2 contents in tomato leaves at 24 hpi. **E**–**G** Effects of SA application on plant defense in TRV-*FAAH1* and its control TRV-0 plants. **E** Representative leaf images for disease symptoms at 3 dpi. **F** Bacterial growth in tomato leaves at 3 dpi. Bar, 2 cm. **G** Changes of NAE18:2 contents in tomato leaves at 24 hpi. **H** Effects of NAE18:2 application on defense-induced SA accumulations at 24 hpi. **I** Effects of NAE18:2 application on the transcript abundance of SA biosynthesis genes (*PAL4* and *PAL6*) at 12 hpi. **J** Effects of NAE18:2 application on the transcript of SA signaling genes (*NPR1*, *PR1b*, and *PR4*) at 12 hpi. TRV-0, VIGS control plants; TRV-*PLDγ*, *PLDγ* gene silencing plants; TRV-*FAAH1*, *FAAH1* gene silencing plants. The data are presented as mean values ± standard deviation (SD); *n* = 3, except for 5 in **C** and **F**. Different letters indicate significant differences between treatments (*P* < 0.05, Tukey’s test).

The signaling molecule SA plays a critical role in plant resistance to (hemi-)biotrophic pathogens [25]. To unveil the underlying molecular crosstalk between NAE and SA signaling in plant defense, we quantified SA contents in VIGS plants after *Pst* DC3000 inoculation. As shown in Fig. 8A, *Pst* DC3000 inoculation led to further induction of SA content in TRV-*PLDγ* plants, whereas *Pst* DC3000-induced SA accumulation was significantly suppressed in TRV-*FAAH1* plants. We also silenced *PLDγ* in SA-abolished NahG plants to further investigate the role of NAE in the SA-mediated defense pathway. Unlike increased defense to *Pst* DC3000 by silencing *PLDγ* in WT control plants, silencing *PLDγ* in NahG plants failed to alleviate severe disease symptoms, and showed similar disease susceptibility to TRV-0 control plants in NahG background (Fig. 8B and C). The analyses of NAE quantification suggested that *Pst* DC3000 failed to suppress NAE18:2 contents in NahG background VIGS plants (Fig. 8D). Consistently, exogenous SA application fully rescued the increased disease susceptibility caused by *FAAH1* silencing (Fig. 8E and F). Also, SA treatment significantly inhibited NAE18:2 accumulation both in TRV-0 and TRV-*FAAH1* plants in response to *Pst* DC3000 infection (Fig. 8G).

Next, we verified the effect of NAE18:2 in the SA defense pathway by exogenous NAE18:2 treatment. Although NAE18:2 did not cause any significant changes in SA contents in mock-inoculated plants, it remarkably suppressed *Pst* DC3000-induced SA accumulation (Fig. 8H). Consistent with the alteration of endogenous SA content, NAE18:2 also compromised the transcript abundance of the SA biosynthesis genes *PAL4* and *PAL6* in response to *Pst* DC3000 inoculation (Fig. 8I). In addition, NAE18:2 suppressed the transcript levels of SA receptor gene *NPR1*, and SA signaling gene *PR1b* and *PR4* at 12 hpi (Fig. 8J). These results suggest that the NAEs suppress tomato defense against *Pst* DC3000 by the antagonism with the SA defense pathway.

## Discussion

**Figure 9:**
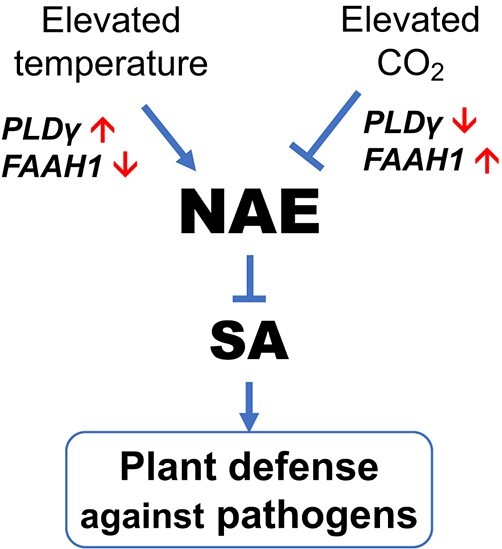
A model of NAE pathway in tomato defense variation in response to changing environments. NAE pathway was regulated by its synthetase PLDγ and hydrolase FAAH1 in plant defense response via inhibition of SA defense signaling. Furthermore, the defense homeostasis of the NAE pathway was influenced by environmental factors, briefly, elevated temperature inhibited, but elevated CO_2_ promoted NAE metabolism resulting in plant defense variation to changing environments.

NAE and its metabolism pathway have been well studied in animals, and their important functions, especially in energy metabolism and pathological immunomodulation, have been well established in animals [31]. In contrast, the role of NAE in plant responses to environmental stimuli and pathogen attacks, as well as the underlying mechanisms have received less attention. Here we provided several lines of evidence demonstrating that the NAE pathway differentially modulated plant defense to different types of pathogens, and the pathway was also crucial for defense variations derived from elevated CO_2_ and temperature potentially via crosstalk with SA-mediated defense signaling (Fig. 9).

### NAEs modulate plant defense differently to different types of pathogens

NAE18:0 was identified in animals as an immunomodulator that participated in noncannabinoid receptor anti-inflammatory signaling pathways [32]. In plants, C18 NAE is the most abundant NAE species regulating plant development but with uncharacterized defense function before this study [1, 3]. Here, we reported that *in planta* exogenous applications of NAE18:0 and NAE18:2 promoted plant defense against the necrotrophic fungal pathogen *B. cinerea* but inhibited resistance to the hemi-biotrophic bacterial pathogen *Pst* DC3000 (Fig. 1). Also, in line with the exogenous effects of NAEs, inoculation with *B. cinerea* significantly induced endogenous accumulation of NAE18:2 and PA in tomato plants (Fig. 5). In agreement with this, previous studies revealed that fungal elicitor xylanase could induce endogenous NAE accumulations [6, 33]. However, *Pst* DC3000 inoculation resulted in suppression or no significant changes of NAE accumulations in tomato (Fig. 5) and Arabidopsis [20], respectively, which was probably attributed to the different systems of plant-pathogen interactions. Interestingly, NAE produced by *Verticillium dahliae* D pathotype could disrupt cotton NAE metabolism, alerting sensitivity to the pathogen [34]. During plant-bacteria communication, the great similarity of NAEs to bacterial quorum-sensing molecules AHLs could function as the reciprocal signal emission from microbes [35]. For insistence, both AHLs [e.g. OdDHL] and NAEs (NAE12:0) were able to inhibit root elongation [15, 36]. Previously, we demonstrated that the exogenous application of AHLs (e.g. DHL, OHHL, and HHL) could induce systemic resistance to *B. cinerea* in tomato, which was consistent with our findings of NAEs [37].

### The key NAE pathway-related genes and their function in plant defense

According to the studies in animals, the formation and metabolism of NAEs are predicted to proceed mainly through PLD- and FAAH-mediated biosynthetic and hydrolysis reactions, respectively [1]. However, the evidence of PLD- and FAAH-mediated metabolism of NAEs was quite limited in plant species. PLD is regarded as an NAE synthase based on the *in vitro* evidence that *Escherichia coli*-expressed recombinant Arabidopsis β/γ type PLD proteins are able to hydrolyze NAPE to NAE and PA [6]. Here we provided *in vivo* evidence that silencing *PLDγ* impaired endogenous NAE18:2 accumulations in tomato plants (Fig. 5). On the other hand, FAAH-mediated NAE degradation has been supported by substantial evidence, although the studies are limited to Arabidopsis only [18–20]. Based on the elevated NAE contents in *FAAH1*-silenced plants (Fig. 5), it can be inferred that FAAH-mediated NAE degradation also takes place in tomato plants.

In this study, the role of tomato PLDs in plant defense was analysed according to the transcript changes in response to pathogen inoculations. Then 3 *PLD* genes (*PLDα1*, *PLDγ*, and *PLDδ1*) were selected based on the significant changes in transcript abundance, especially *PLDγ* with the highest induction to *B. cinerea* and intense suppression to *Pst* DC3000, respectively (Fig. 2C). The results of loss- and gain-function studies further demonstrated that PLDγ showed dual effects on plant defense that largely varied with the pathogen types; briefly, PLDγ induced defense against necrotrophic *B. cinerea* but it suppressed the resistance to *Pst* DC3000 (Figs 3 and 4). Before this report, our knowledge of γ type PLD in plant defense response was based on the only study showing that Arabidopsis PLDγ1 was induced at the protein level and recruited to the plasma membrane during effector-triggered immunity [38]. Several studies have demonstrated the distinct roles of plant β type PLDs in defense response, for example, rice PLDβ functions as a negative regulator to biotrophic and hemi-biotrophic pathogens *P. grisea* and *X. oryzae* pv. *oryzae*, respectively [8], and Arabidopsis PLDβ1 enhances defense against *B. cinerea* but suppresses resistance to both virulent and avirulent *Pst* DC3000 strains [9]. Because PLDβ/γ are close clusters in the phylogenetic tree and crucial for NAE biosynthesis, it is reasonable to imply PLDβ/γ in similar roles in plant defense. In addition, considering these dual effects of PLDβ/γ on plant defense response to different pathogens, it is highly plausible that PLD-mediated defense response is associated with different infection modes of necrotrophic and (hemi-)biotrophic pathogens.

Unlike a unique FAAH in Arabidopsis, tomato embraces 4 FAAH encoding genes. However, only *FAAH1* out of four tomato FAAH genes exhibited distinctly changed transcript abundance when plants were subjected to pathogen inoculation, i.e. *FAAH1* was suppressed by *B. cinerea,* but largely induced by *Pst* DC3000 (Fig. 2D). Due to the opposite roles of PLD and FAAH in the NAE pathway, the *FAAH1* transcripts also consistently showed the opposite trends compared to *PLDγ* in response to pathogen inoculation. In accordance with this, the transcript abundance of Arabidopsis *FAAH* was also highly induced by *Pst* DC3000 inoculation [20]. Surprisingly, in spite of high *Pst* DC3000-induced *FAAH*, Arabidopsis *FAAH* overexpression lines are comprised in the defense response to *Pst* DC3000, while its *faah* knockout mutants show similar disease resistance like wild type plants [20]. The present study showed that tomato FAAH1 differentially regulated plant defense by increasing resistance against *Pst* DC3000 but suppressing defense to *B. cinerea* (Figs 3 and 4). This conclusion was also indirectly supported by the findings of PLDγ in plant defense, because FAAH1 and PLDγ acted in opposite directions regarding NAE accumulation. In addition, the disease phenotype in *FAAH1*-silenced plants caused by these two types of pathogens largely coincided with the effect of exogenous NAE application. Taken together, these findings suggested that PLDγ and FAAH1 cooperate in the NAE pathway to differentially regulate plant defense to hemi-biotrophic and necrotrophic pathogens.

### NAE pathway is associated with plant defense variations in response to different environments

As a consequence of increasing anthropogenic activities, the atmospheric CO_2_ concentrations and temperatures are increasing, which greatly affect plant growth and defense responses [39, 40]. Unlike the general stimulatory effect of elevated CO_2_ on plant defense, elevated temperatures have been demonstrated to promote disease susceptibility in compatible plant-pathogen interactions [30, 41, 42]. Notably, both high CO_2_ and high temperatures can trigger plant lipid signaling and modifications [28, 43, 44]. Here, we provided evidence that NAE as the lipid-derived molecules and its pathway could be affected by changing environments, leading to defense variations under elevated CO_2_ and elevated temperature.

According to the transcript levels of NAE-related genes, we found that elevated CO_2_ suppressed NAE accumulations in response to pathogen infection, whereas elevated temperature played the opposite role in the NAE pathway compared with elevated CO_2_ (Figs 7 and 8). Disruption of NAE accumulations somehow impaired high-CO_2_ induced plant defense but alleviated high temperature-induced disease susceptibility (Figs 7 and 8). Notably, the downstream hydrolysis product of NAEs, FFAs such as FFA 18:0 and FFA 18:2 can be induced by high-CO_2_ in *Chlorella vulgaris* [45]. Studies in Arabidopsis also reported that increased levels of polyunsaturated FFA led to enhanced defense against avirulent bacterial pathogens, whereas FFA biosynthetic mutants exhibited increased resistance to high temperature [46, 47]. Overall, the lipid-derived NAE pathway seems sensitive to the environmental changes in response to pathogen attacks and is crucial for environment-mediated plant defense variation.

### The relationship between NAE pathway and SA defense signaling

SA occupies the center of the resistance network against pathogens, especially for (hemi-)biotrophic pathogens. In plants, pathogen-induced SA synthesis via the phenylpropanoid pathway triggers the master regulator NPR1-promoted transcriptional reprogramming of the defense genes, such as PR genes [22]. It was previously reported that the NAE structural analog AHLs (e.g. oxo-C8-HSL and oxo-C14-HSL) primed resistance to *Pst* DC3000 via the SA defense signaling pathway [48, 49]. However, we proposed that NAE functioned upstream and negatively regulated SA-mediated defense signaling. This conclusion relied on several lines of evidence as follows. First, *Pst* DC3000-induced SA accumulation was further enhanced in TRV-*PLDγ* plants but largely compromised in TRV-*FAAH1* plants (Fig. 8A). In agreement with this study, Arabidopsis *pldβ1* mutants also exhibited higher levels of SA and enhanced activation of SA-regulated defense genes in response to *Pst* DC3000 virulent and avirulent strains [9], and *AtFAAH* overexpression resulted in a higher level of pathogen-induced free SA content than that of Col-0 control plants [20]. Second, the increased defense by silencing *PLDγ* was abolished and high NAE18:2 accumulation was maintained in SA-defective NahG plants, while the SA application was able to suppress endogenous NAE18:2 contents and fully rescue more severe disease symptoms due to *FAAH1* silencing (Fig. 8B–G). Third, NAE18:2 negatively regulated the SA defense pathway through inhibiting pathogen-induced SA accumulation and suppressing the induction of SA biosynthetic *PAL* genes and SA signaling marker genes in response to pathogen infection (Fig. 8H–J). Interestingly, *Pst* DC3000-induced SA accumulation and signaling were inhibited by elevated temperature but activated by elevated CO_2_ in plant defense response [25, 30].

In conclusion, the data presented in this article demonstrate the distinct roles of NAEs and NAE pathway in plant defense to different types of pathogens underlying the crosstalk with the SA defense signal. In response to changing environments, tomato plants manipulated NAEs and NAE pathway to respond with defense variations in different environmental conditions. Thus, manipulation of NAEs and its metabolism appears to be an appealing strategy for potential plant protection from different pathogens in various environmental conditions.

## Materials and methods

### Plants, pathogens, and treatments

The wild-type tomato (*Solanum lycopersicum* L.) cv. Moneymaker and phytohormone NahG mutant were used in this study. The NahG plants artificially express an SA hydroxylase that causes endogenous SA degradation. Tomato plants were grown in trays and then transferred to pots containing a mixture of peat and vermiculite (3:1, v/v) in plant growth chambers, under 14/10 h (day/night) photoperiod, 500 μmol m^−2^ s^−1^ photosynthetic photon flux density, and 24/21°C (day/night) air temperatures.

Stearoyl ethanolamide (NAE18:0) (Sigma-Aldrich, St. Louis, MO, USA), linoleyl ethanolamide (NAE18:2) (Sigma-Aldrich, St. Louis, MO, USA) were dissolved in ethanol, and then diluted with dH_2_O to 80 μM working solution. Approximately 5-week-old tomato plants at about the five- to the six-leaf stage were used for each experiment. Tomato leaves were sprayed with fresh solutions of 80 μM NAE18:0, 80 μM NAE18:2, or corresponding ethanol solution as control, and 2 mM SA (Aladdin, China) or dH_2_O as control once daily on both the adaxial and abaxial surfaces. Two days after the reagent treatment, the plants were subjected to pathogen inoculations.

Fungal *B. cinerea* B05–10 strain, bacterial *Pst* DC3000 were used for pathogen inoculation in this study. The steps of pathogen cultivation and isolation were performed according to our previous work [25]. For *B. cinerea* inoculation, tomato plants were uniformly sprayed with fungal spore suspended in dH_2_O containing 0.01% Tween-20 (v/v), or 2.5 μL of fungal spore suspension was dipped on the upper surface of the detached leaves. *Pst* DC3000 inoculation was performed by spraying the bacteria at the concentration of OD_600_ = 0.05 suspended in 10 mM MgCl_2_ and 0.02% Silwet (v/v) on the whole leaves of tomato. The plants were sprayed with corresponding suspension buffer as mock inoculation.

To investigate the plant defense in response to changing environments, half of the environmental setting of respective growth chambers was changed 2 d before pathogen inoculation, while the rest of the chambers were maintained at previous environment conditions mimicking the ambient status. For elevated CO_2_ treatment, the chamber setting of CO_2_ concentration was maintained at 400 μmol mol^−1^ or changed to 800 μmol mol^−1^, corresponding to the ‘ambient CO_2_’ and the ‘elevated CO_2_’ conditions, respectively. For elevated temperature treatment, the chamber temperature setting was kept at 24/21°C or increased to 30/27°C (day/night) air temperature, corresponding to the ‘ambient temperature’ and the ‘elevated temperature’, respectively.

### Virus-induced gene silencing and transient overexpression

For the virus-induced gene silencing (VIGS) assay, the *PLD* family genes and *FAAH1* were amplified from tomato cDNA by PCR using specific primers as listed in Table S1 (see online supplementary material). Then, the target gene fragments were ligated into the *Tobacco rattle virus*-based vector pTRV2, and then were electroporated into *Agrobacterium tumefaciens* GV3101 strain. VIGS was performed based on Agrobacterium-mediated transformation as described previously [50]. The quantitative real-time (qRT)-PCR was performed to select the plants with over 70% silencing efficiency for the following experiments.

Transient overexpression of PLD family genes and *FAAH1* were performed according to Agrobacterium-mediated transient overexpression in tomato [24]. Briefly, the full-length CDS of the target *PLD* genes and *FAAH*1 were cloned into the pAC402 vector driven by the 35S promoter and with an HA epitope tag in the C-terminus. The specific primers for PCR amplification were listed in Table S1 (see online supplementary material). Then, the plasmids were also transformed into *A. tumefaciens* C58C1 strain. *A. tumefaciens*-mediated plasmid transformation was performed in leaves of 4-week-old tomato plants. After two-day protein expression, the tomato leaves were subjected to pathogen inoculation.

### Disease symptom assays

Disease symptoms were evaluated by quantifying pathogen *Actin* mRNA accumulation for *B. cinerea* infection, and analysed by bacterial growth for *Pst* DC3000 infection [25]. In addition, trypan blue staining and chlorophyll fluorescence measurements were also performed to support the disease symptom evaluation.

Chlorophyll fluorescence was measured with a PAMsetup (IMAG-MAXI; Heinz Walz, Germany). The photochemical quantum efficiency of PSII (Φ_PSII_) was analysed based on the previous studies [25, 51].

For the ROS production assay, more than four leaves from 5-week-old tomato plants were excised into at least 12 leaf discs of 0.25 cm^2^ and incubated in a 96-well plate with 100 μL dH_2_O overnight. 100 μL of reaction solution containing 100 μM luminal and 20 μg mL^−1^ horseradish peroxidase (Sigma-Aldrich, St. Louis, MO, USA) supplemented with 100 nM flg22 was added to each well. The measurement was performed by the Centro LB 960 plate luminometer (Berthold Technologies) immediately after adding the reaction solution. The ROS production was presented as means of relative light units (RLU).

### RNA isolation and transcript level analysis

Total RNA from tomato leaves was extracted by the RNAprep Pure Plant Kit (Tiangen Biotech, China) following the manufacturers’ instructions. RNeasy Mini Kit (Qiagen, Germany) was used to exclude the residual genomic DNA. After measuring the RNA concentration by the NanoDrop 2000 system (Thermo, USA), the ReverTra Ace qPCR RT Kit (Toyobo, Japan) was used for reverse transcription with approximately 1 μg of total RNA as the template. Then, qRT-PCR was performed based on the previously described methods using the LightCycler 480 Real-Time PCR System (Roche, Switzerland) [26]. The relative expression levels were normalized to the expression level of the tomato *ACTIN2* and *UBI3* genes. The specific primers for qRT-PCR were listed in Table S2 (see online supplementary material).

### Lipid extraction and quantification

Lipid extraction was performed based on a reported study with minor modifications [51]. Approximately 0.5 g of tomato leaves were collected and quickly ground in liquid nitrogen. Next, the samples were added to 2 mL of hot 2-propanol (70°C) with 40 μL 500 ng mL^−1^ NAE18:2-D4 (Shanghai ZZBIO Co., China), and heated at 70°C for 30 min. 1 mL of chloroform and 300 μL of dH_2_O were added to the mixtures for the overnight lipid extraction at 4°C. After centrifuging at 10000 rpm for 5 min at 4°C, the separated organic top layer was transpipetted to clean centrifuge tubes, 1 mL of chloroform and 2 mL of 1 M KCl were added to induce phase separation. After aspirating off the aqueous layer, the organic layer was washed two times with 2 mL of 1 M KCl and one time with 2 mL of dH_2_O. The organic phase was collected and dried by nitrogen gas. 300 μL 50% methanol was added, vigorously swirled, centrifuged for next LC–MS analysis.

The lipid samples were subjected to the LC–MS analyses on an Agilent 6460 triple quadrupole mass spectrometer (Agilent Technologies, USA) with an electrospray ionization (ESI) source. Samples were analysed using a Zorbax SB C18 column (150 × 2.1 mm, 3.5 μm) with 2 mM ammonium acetate in 0.1% formic acid as solvent A and 100% methanol as solvent B at a flow rate of 0.3 mL/min. The gradient program was as follows: (i) 2 min of 50% A and 50% B; (ii) 3 min of 15% A and 85% B; (iii) 6 min of 4% A and 96% B; and (iv) 6 min of 50% A and 50% B. Then, the ESI source in positive ion multiple-reaction monitoring (MRM) mode was used to complete the mass spectrometric detection of specific lipid molecules. The following MS parameters were used to detect NAE18:2 (precursor ion, product ion): NAE18:2 (324.3, 62.2), and NAE18:2-D4 (328.2, 66.2).

### PA and SA analysis

PA was extracted from tomato leaves and analysed by the Plant PA ELISA KIT (Shanghai Enzyme-linked Biotechnology Co., China) following the manufacturers’ instructions. Endogenous SA was extracted from tomato leaves and quantified by HPLC–MS/MS (Agilent 6460; Agilent Technologies, USA) with a final concentration of 100 ng mL^−1^ D4-SA (OlChemlm, Czechia) as internal standards using previously reported procedures [50].

### Statistical analysis

For each individual experiment, at least three independent biological replicates were sampled and each biological replicate consisted of an independent sample that was pooled of two leaves from one individual plant. The experiments were independently performed at least two times. The collected data were subjected to analysis of variance using SAS software, version 8 (SAS Institute, USA), and means were compared using Tukey’s test at the 5% level.

## Acknowledgements

This work was supported by the Key Research and Development Program of Zhejiang Province (2021C02040), the National Natural Science Foundation of China (32172650, 31902097), the Natural Science Foundation of Zhejiang Province (LR19C150001), and the Starry Night Science Fund of Zhejiang University Shanghai Institute for Advanced Study (SN-ZJU-SIAS-0011).

## Author contributions

K.S. conceived and designed the research; Z.H., J.S., S.F., and S.S. performed the experiments; X.W. provided technical/intellectual support; Z.H. and K.S. wrote the article with contributions from other authors.

## Data availability

All data supporting the findings of this study are available within the paper and within its supplementary data published online.

## Conflict of interest

The authors declare that they have no conflict of interest.

## Supplementary data

Supplementary data is available at *Horticulture Research* online.

## Supplementary Material

Web_Material_uhac242
