# Supplementary material for: Plant N-acylethanolamines play a crucial role in defense and its variation in response to elevated CO2 and temperature in tomato
Source: Hortic Res. 2022 Oct 26;10(1):uhac242. doi: 10.1093/hr/uhac242 (PMC10108025; doi:10.1093/hr/uhac242)
Supplement: Web_Material_uhac242 [file web_material_uhac242.zip › Supplemental Data.pdf]

Supplementary data

Article title: Plant *N*-acylethanolamines play a crucial role in defense and its variation in response to elevated CO_2 and temperature in tomato

Authors: Zhangjian Hu, Junying Shi, Shuxian Feng, Xiaodan Wu, Shujun Shao, Kai Shi

The following supporting data are available for this article:

Fig. S1. Alignment of partial amino acid sequences of FAAHs from *Arabidopsis thaliana* (At), *Solanum lycopersicum* (Sl), and *Medicago truncatula* (Mt).

Fig. S2. Transcript abundance of homologous genes in target gene-silenced plants.

Table S1. PCR primer sequences used for vector construction.

Table S2. List of primer sequences used for qRT-PCR analysis.

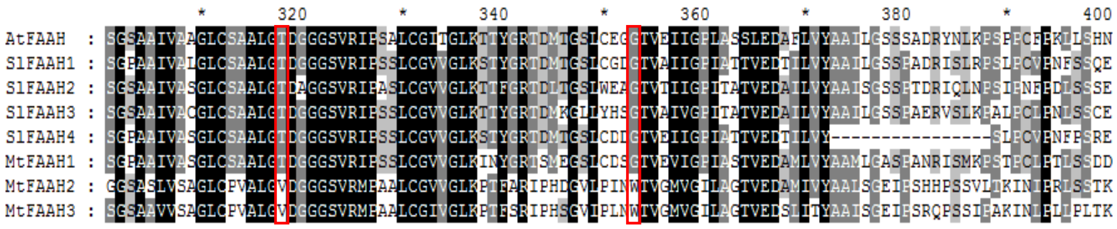

Fig. S1. Alignment of partial amino acid sequences of FAAHs from *Arabidopsis thaliana* (At), *Solanum lycopersicum* (Sl), and *Medicago truncatula* (Mt). The residues are named and numbered based on their identity and position in AtFAAH. Thr^{319} and Gly^{354} are conserved in group I FAAH, but are substituted by conserved Val and Trp, respectively, in group II FAAH.

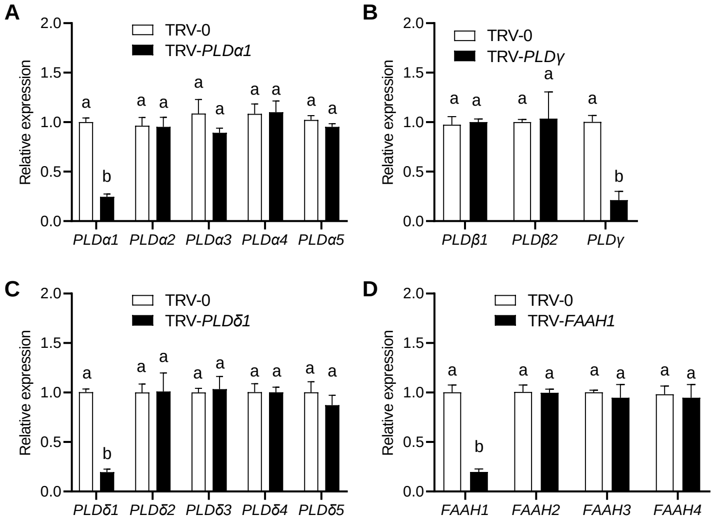

Fig. S2. Transcript abundance of homologous genes in target gene-silenced plants. (A) Relative expression of *PLDα1-PLDα5* in TRV-*PLDα1* and TRV-0 plants. (B) Relative expression of *PLDβ1*, *PLDβ1*, and *PLDγ* in TRV-*PLDγ* and TRV-0 plants. (C) Relative expression of *PLDδ1-PLDδ5* in TRV-*PLDδ1* and TRV-0 plants. (D) Relative expression of *FAAH1-FAAH4* in TRV-*FAAH1* and TRV-0 plants.

Table S1. PCR primer sequences used for vector construction

Vector	Primers
pTRV2-*PLDγ*	F:5’-CGCGGATCCGCTGTGGGAATTTCTGTA -3’<br>R: 5’-CCGGAATTCTACTTTGCCACCTCTTCT -3’
pAC402- *PLDγ*	F: 5’- TCCCCCGGGGATTCTTGAGTGATTACCTGT -3’<br>R: 5’- CGACGCGTGATGGTGAGATTTTCTTGAAC -3’
pTRV2-*PLDδ1*	F:5’-TGCTCTAGACGGAAACCTCAAACTGGA -3’<br>R: 5’-CCGGAATTCAAGGGATACGGAAATGCT -3’
pAC402- *PLDδ1*	F: 5’- TGCTCTAGACTATCTTCAAACACAACCTT -3’<br>R: 5’- CGACGCGTGTAGTCAAAACATCTGGGAT -3’
pTRV2-*PLDα1*	F:5’- CCGGAATTCATGGCTCCGATTCTGCTT -3’<br>R: 5’- CGCGGATCCGGAAAGACTCATACCACCT -3’
pAC402-*PLDα1*	F: 5’-TCCCCCGGGATGGCTCCGATTCTGCTT-3’<br>R: 5’-CGACGCGGGTAGTGAGGATAGGAGG -3’
pTRV2-*FAAH1*	F:5’-CGCGGATCCCTGACAAGTGCGGGAATG -3’<br>R:5’-CCGGAATTCCTGCTGCTGTAAATGAACG -3’
pAC402-*FAAH1*	F: 5’- TGCTCTAGAATTGTTGTGTGTGATAAATTGAG -3’<br>R: 5’- CGACGCGGTTCCCTTTCAGAATGTCATAA -3’

The restriction enzyme sites were underlined.

Table S2. List of primer sequences used for qRT-PCR analysis

Gene	Forward primer (5'-3')	Reverse primer (5'-3')
PLDα1	CAATGGATGGTGCAAGAGAC	TTCCATTCACCTTCCTGACA
PLDα2	GAAATAGCCATGGGAGCCTA	ACAATGCCATTCTGAAACCA
PLDα3	GCACTGTGGTACGAGCACTT	CAGGCAAATCATGAACAAGG
PLDα4	GTGAACCGCCAGAACCTAAT	CTCTTGCACCATCCATTGAC
PLDα5	GTCAATGGATGGTGCAAGAG	GCCATGCCATTTACTTTCCT
PLDβ1	AATGTGGCCAGAGGGTAATC	ATGAATTCTCAAGCCCAACC
PLDβ2	AGGCATCTTCTGTGGTCCAA	ACGTCCATGTTCGGTAGGTT
PLDγ	TGAACATCCAGAGAGCCTTG	GGTGGCCTTTCATTTCAGTT
PLDδ1	TATAATGGGCTCTGCCAACA	TGTCCATGCATAATGAGGCT
PLDδ2	ATTGATGGACCTGCTGCATA	AATTCCCTCCACTTTGTTGC
PLDδ3	AATCGGAGGAAGGTGTGAGG	TACTGCTCGCATAACGAGGT
PLDδ4	CCGGATGGTGACCAAGTAGT	AGCCAGGTGTATGCTTCTGT
PLDδ5	GCAGCATACATTGTGGTTCC	AAGAGCCTCTGCCACAATTT
FAAH1	CAAATTCGACGAGAAGCTGA	GATGCTGACAACAGCAGGTT
FAAH2	ACTTATTGGTCGTCCGTGGT	AACCGTTCCCTTTCAAGATG
FAAH3	CTCAGGCTCTGCAGCTATTG	ACACCACAAAGGGAAGAAGG
FAAH4	TGCAGCTTTAGGGACAGATG	CCCTGTCATGTCAGTTCGAC
NPR1	GGGAAAGATAGCAGCACG	GTCCACACAAACACACACATC
PR1b	TAGTCTGGCGCAACTCAGTC	TGCAAGAAATGAACCACCAT
PR4	TAGTCTGGCGCAACTCAGTC	TGCAAGAAATGAACCACCAT
PAL4	CTCTTCGTGGCACAATCACT	TCCTCAGCATTCAACGTCTC
PAL6	TGGTAGGCCTAATTCCAAGG	TGCAAATCCTTCCTTAGGCT
ACTIN2	TGTCCCTATTTACGAGGGTTATGC	CAGTTAAATCACGACCAGCAAGAT
UBI3	GCCGACTACAACATCCAGAAGG	TGCAACACAGCGAGCTTAACC
B. cinerea ACTIN	ACTCATATGTTGGAGATGAAGCGCA	AATGTTACCATACAAATCCTTACGGA
