# Supplementary material for: Plant N-acylethanolamines play a crucial role in defense and its variation in response to elevated CO2 and temperature in tomato
Source: Hortic Res. 2022 Oct 26;10(1):uhac242. doi: 10.1093/hr/uhac242 (PMC10108025; doi:10.1093/hr/uhac242)
Supplement: Web_Material_uhac242 [file web_material_uhac242.zip › Fig. S1.pdf]

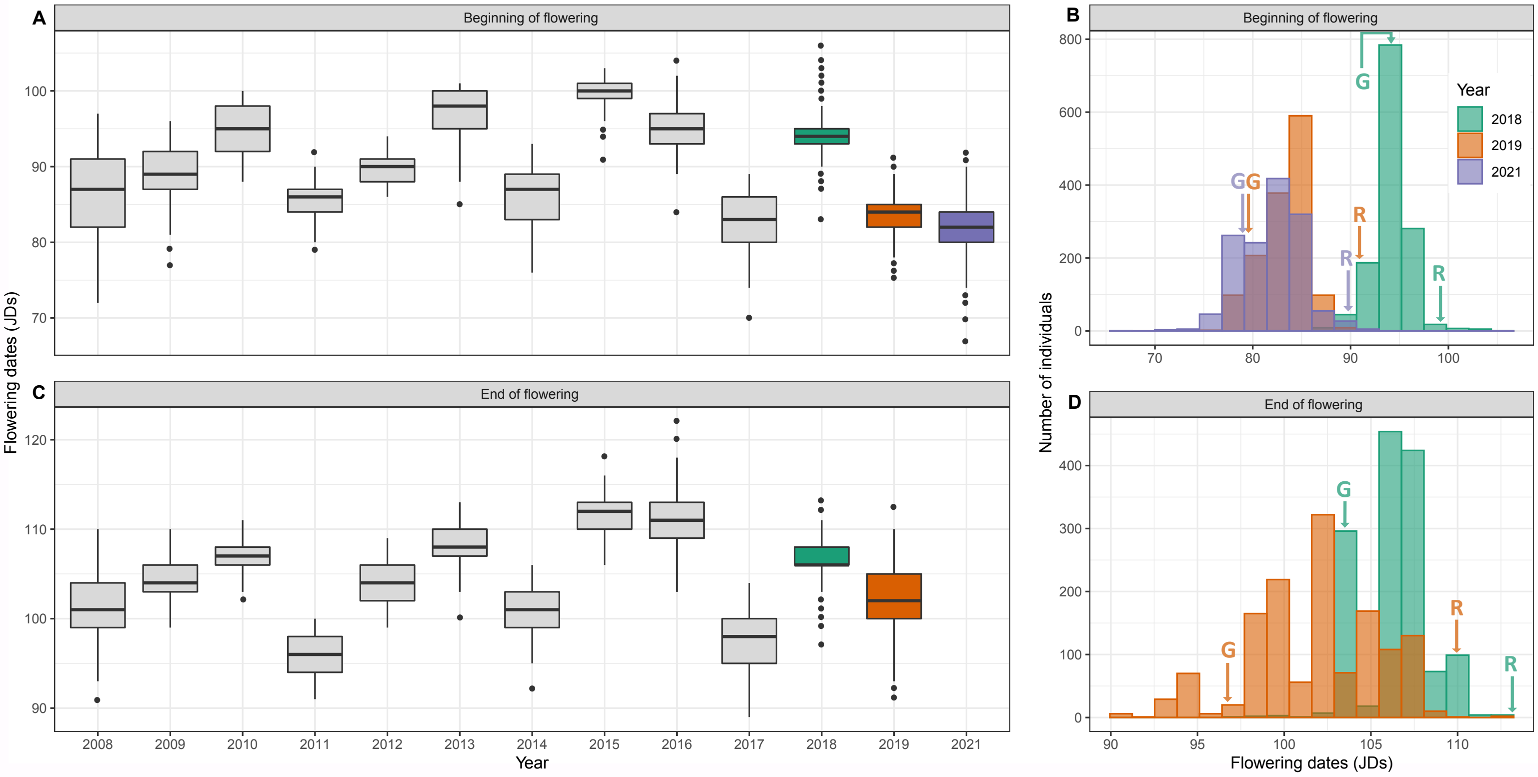

Fig. S1 Distribution of flowering date in Population #1 (from 2008 to 2017, in A and C) and Population #2 (in 2018, 2019 and 2021, in B and D).

Beginning of flowering is scored in Julian days (JDs) across ten years in Population #1 (in grey in A) and three years in Population #2 (2018 in green, 2019 in orange and 2021 in purple, in A and B). End of flowering is scored in JDs across ten years in Population #1 (in grey in C) and two years in Population #2 (2018 in green and 2019 in orange, in C and D). Data for parental cultivars 'Regina' (R) and 'Garnet' (G) in 2018, 2019 and 2021 is indicated by arrows in B and D.
