# Supplementary material for: Plant N-acylethanolamines play a crucial role in defense and its variation in response to elevated CO2 and temperature in tomato
Source: Hortic Res. 2022 Oct 26;10(1):uhac242. doi: 10.1093/hr/uhac242 (PMC10108025; doi:10.1093/hr/uhac242)
Supplement: Web_Material_uhac242 [file web_material_uhac242.zip › Fig. S2.pdf]

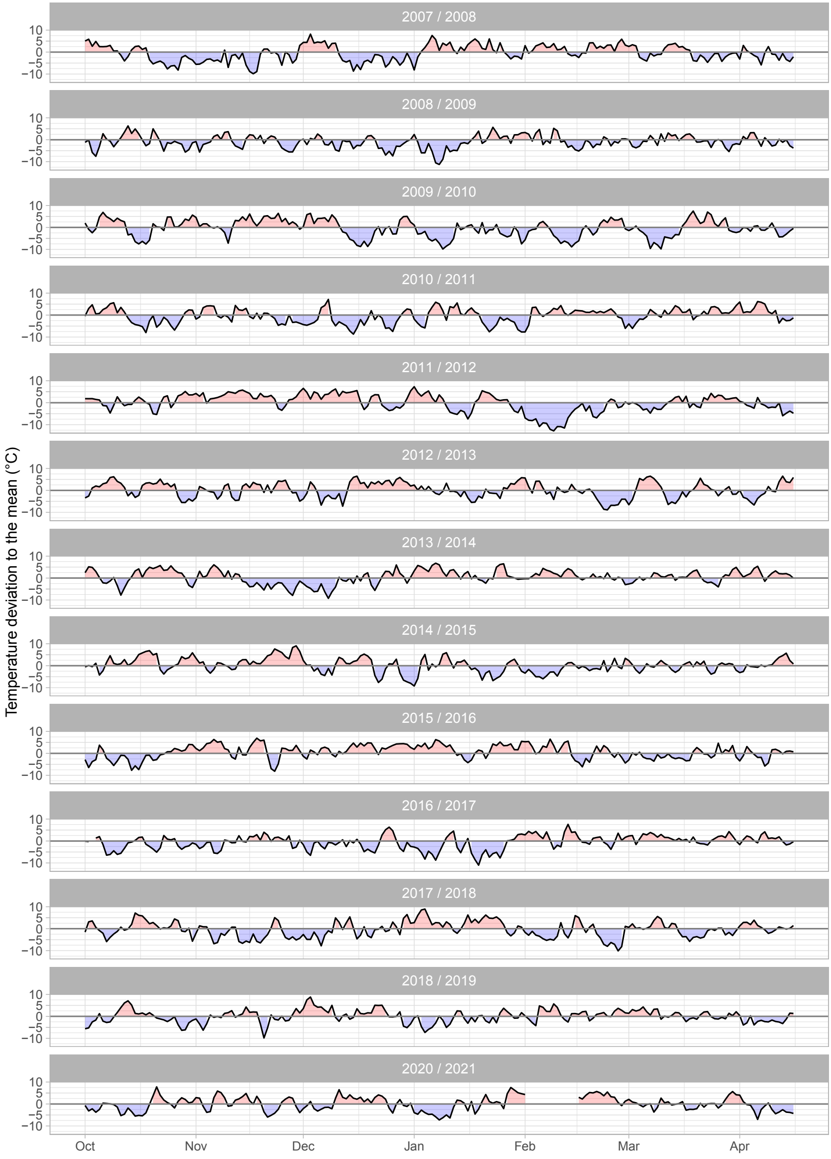

Fig. S2 Temperature deviation to the mean between 2008 and 2021 for the period spanning from October to April. The mean was calculated using temperature data from 2007 to 2021.
