# Supplementary material for: Plant N-acylethanolamines play a crucial role in defense and its variation in response to elevated CO2 and temperature in tomato
Source: Hortic Res. 2022 Oct 26;10(1):uhac242. doi: 10.1093/hr/uhac242 (PMC10108025; doi:10.1093/hr/uhac242)
Supplement: Web_Material_uhac242 [file web_material_uhac242.zip › Fig. S3.pdf]

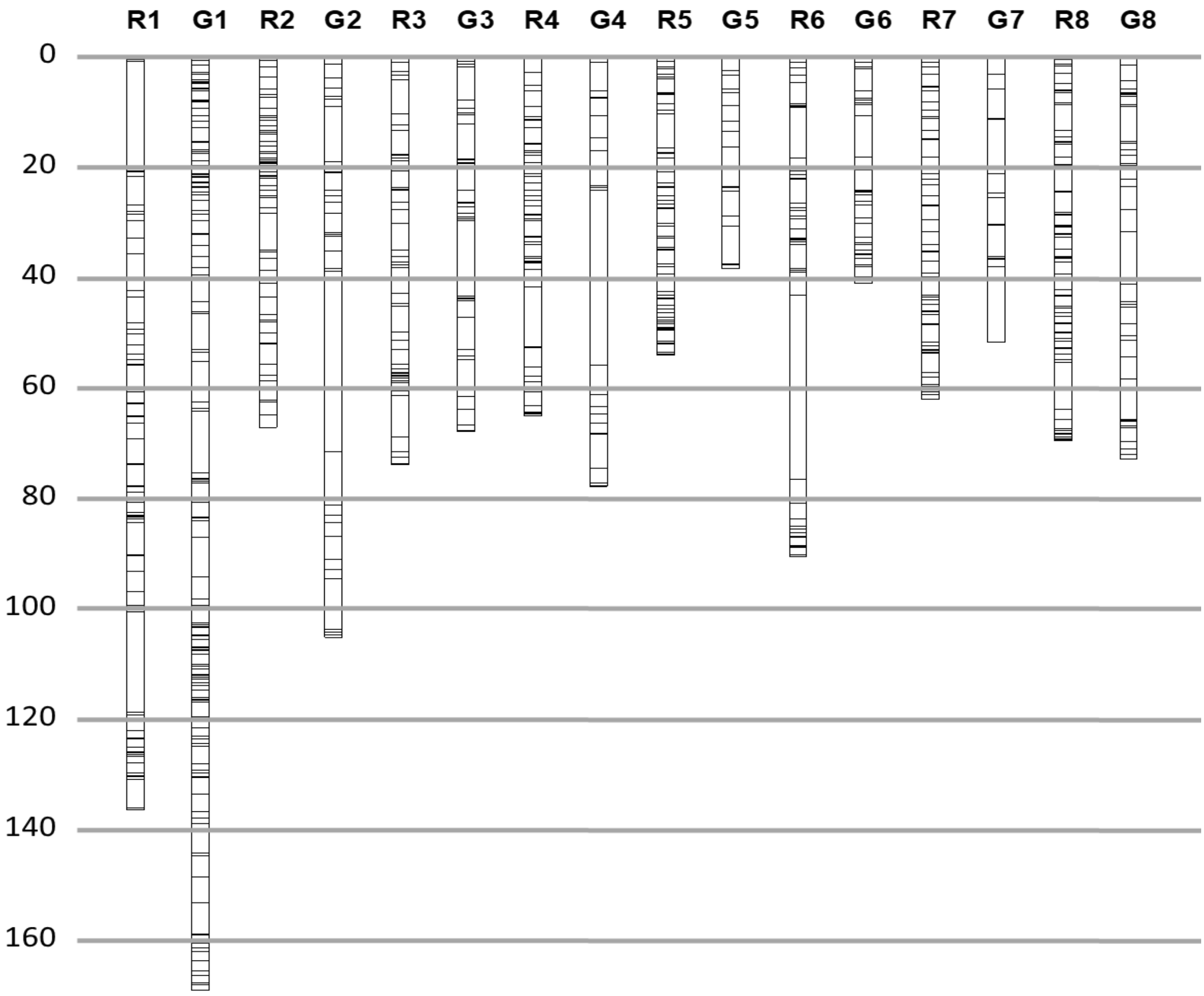

Fig. S3 Parental genetic linkage maps of 'Regina' (R1 to R8) and 'Garnet' (G1 to G8) using 454 ‘Regina’ x ‘Garnet’ hybrids from the Population #2. The Y axis represents the genetic lenght in centimorgans (cM).
