# Supplementary material for: Plant N-acylethanolamines play a crucial role in defense and its variation in response to elevated CO2 and temperature in tomato
Source: Hortic Res. 2022 Oct 26;10(1):uhac242. doi: 10.1093/hr/uhac242 (PMC10108025; doi:10.1093/hr/uhac242)
Supplement: Web_Material_uhac242 [file web_material_uhac242.zip › Fig. S4.pdf]

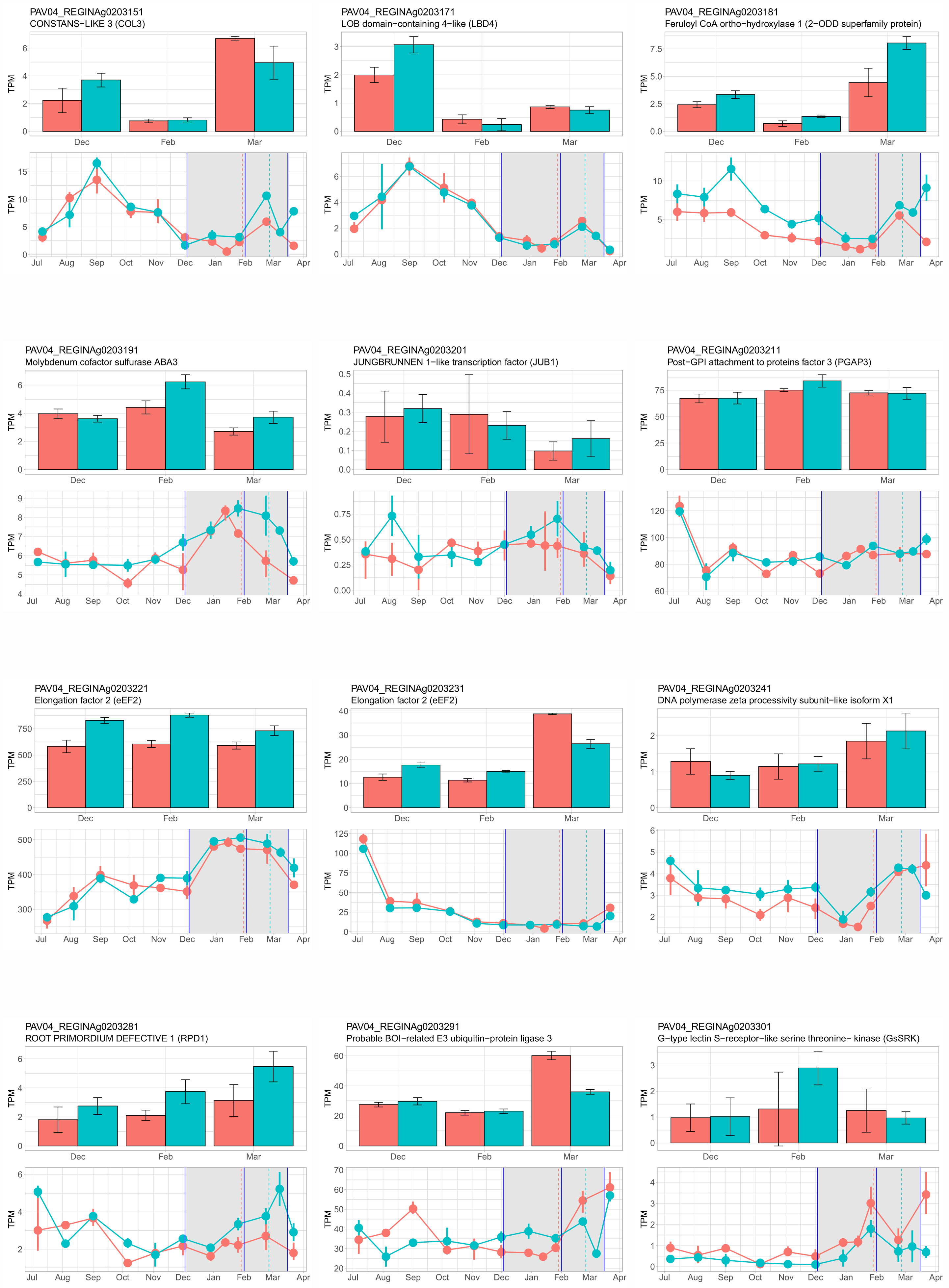

Fig. S4 Expression profiles of 46 predicted candidate genes located within the 'Regina' LG 4 QTL.
For each gene, two expression profiles are presented: the upper one is from the 2009/2010 RNA-seq analysis, across three sampling dates (3 December 2009, 1 February 2010 and 16 March 2010); the lower one is from the 2015/2016 RNA-seq analysis, between July 2015 and April 2016. Expression levels are measured in transcripts per million (TPM) in cultivars 'Regina' in blue and 'Garnet' in red. In the lower profile, the period colored in grey is the period in common with the first RNA-seq analysis, from December to March, and vertical blue lines correspond to the three sampling dates of the first analysis (3 December, 1 February and 16 March) (information added for potential qualitative comparison). Dotted lines correspond to the dormancy release dates for 'Garnet' (in red) and 'Regina' (in blue) in 2015/2016.

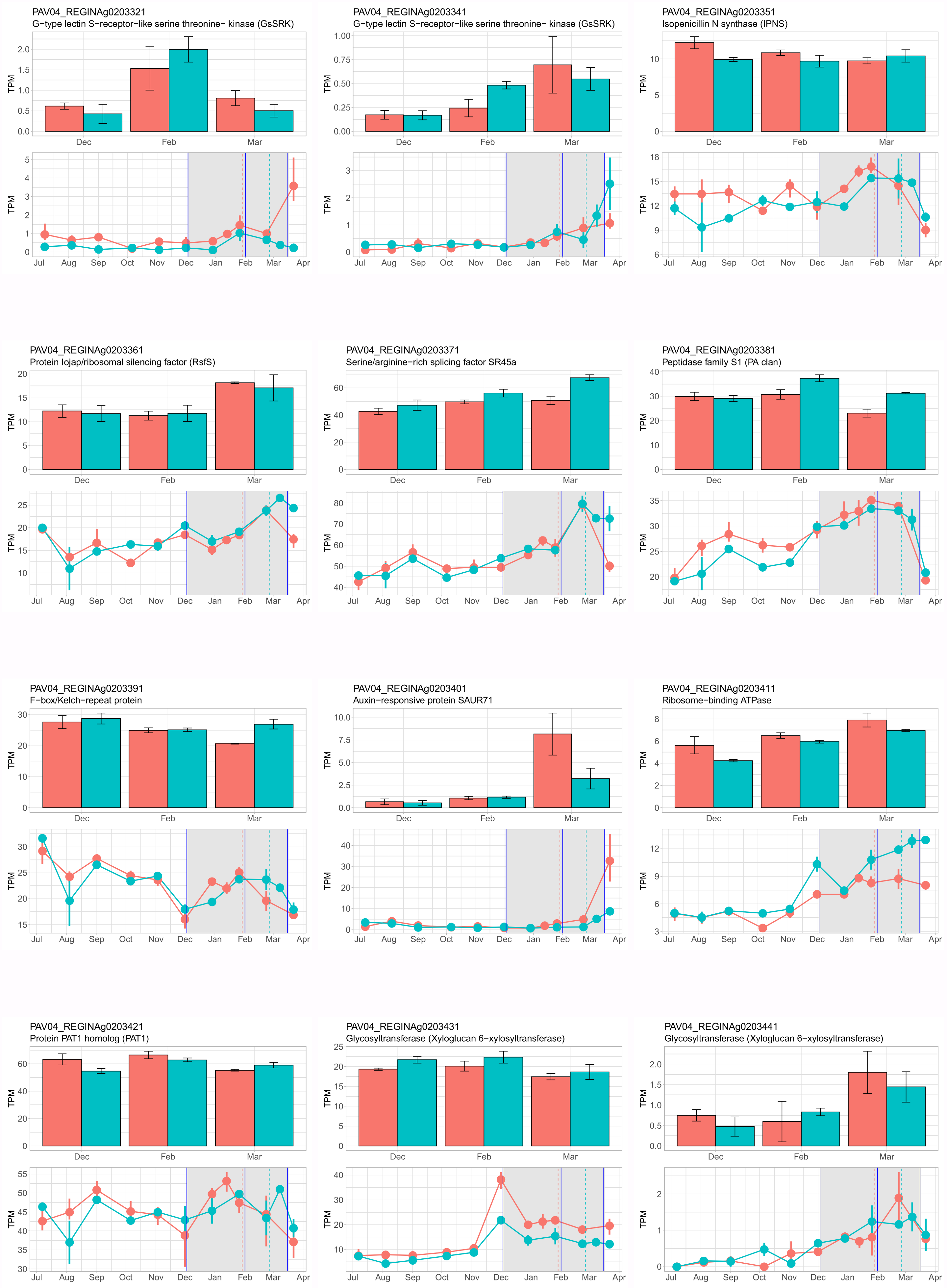

Fig. S4 Continued.
For each gene, two expression profiles are presented: the upper one is from the 2009/2010 RNA-seq analysis, across three sampling dates (3 December 2009, 1 February 2010 and 16 March 2010); the lower one is from the 2015/2016 RNA-seq analysis, between July 2015 and April 2016. Expression levels are measured in transcripts per million (TPM) in cultivars 'Regina' in blue and 'Garnet' in red. In the lower profile, the period colored in grey is the period in common with the first RNA-seq analysis, from December to March, and vertical blue lines correspond to the three sampling dates of the first analysis (3 December, 1 February and 16 March) (information added for potential qualitative comparison). Dotted lines correspond to the dormancy release dates for 'Garnet' (in red) and 'Regina' (in blue) in 2015/2016.

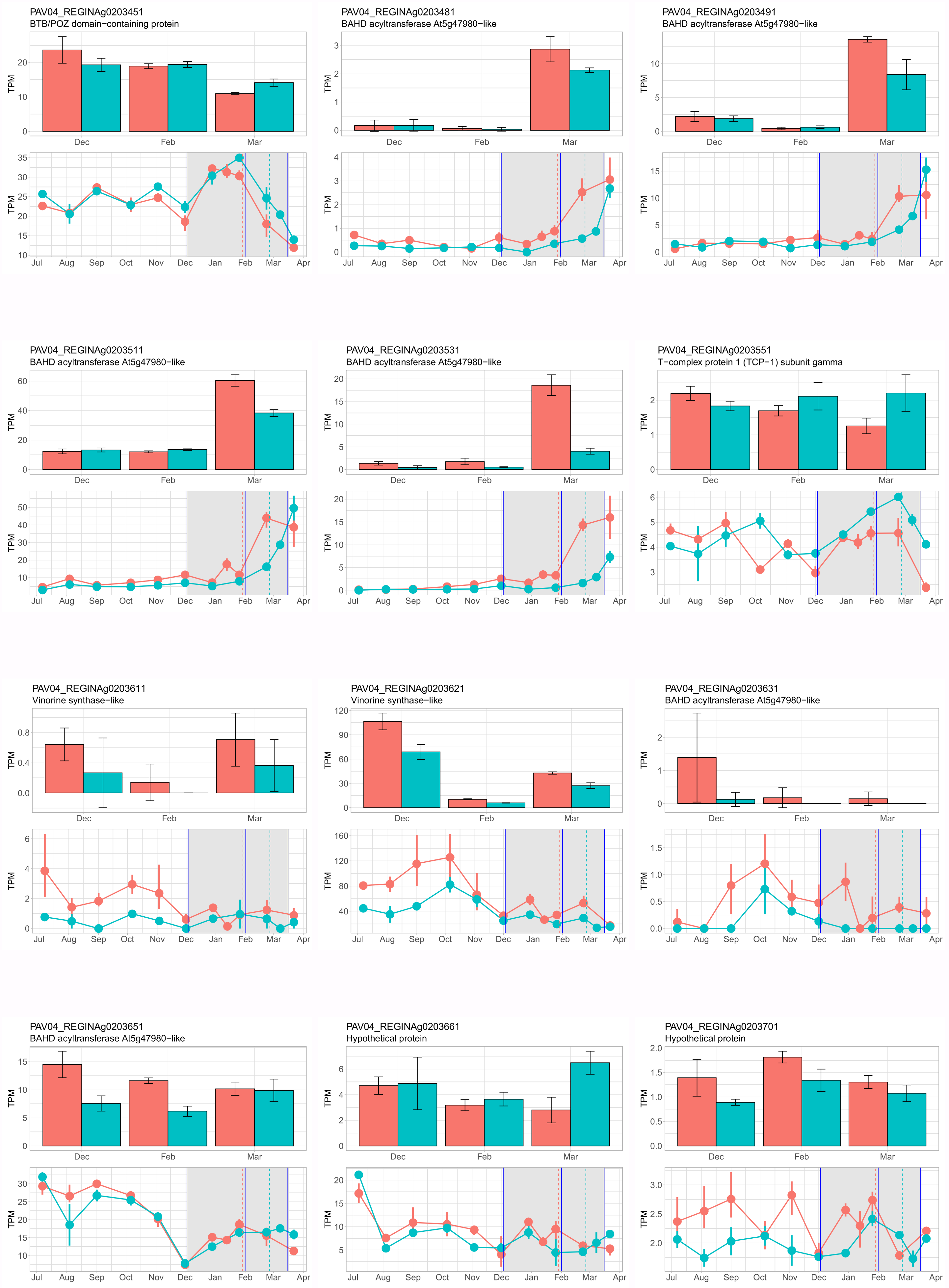

Fig. S4 Continued.
For each gene, two expression profiles are presented: the upper one is from the 2009/2010 RNA-seq analysis, across three sampling dates (3 December 2009, 1 February 2010 and 16 March 2010); the lower one is from the 2015/2016 RNA-seq analysis, between July 2015 and April 2016. Expression levels are measured in transcripts per million (TPM) in cultivars 'Regina' in blue and 'Garnet' in red. In the lower profile, the period colored in grey is the period in common with the first RNA-seq analysis, from December to March, and vertical blue lines correspond to the three sampling dates of the first analysis (3 December, 1 February and 16 March) (information added for potential qualitative comparison). Dotted lines correspond to the dormancy release dates for 'Garnet' (in red) and 'Regina' (in blue) in 2015/2016.

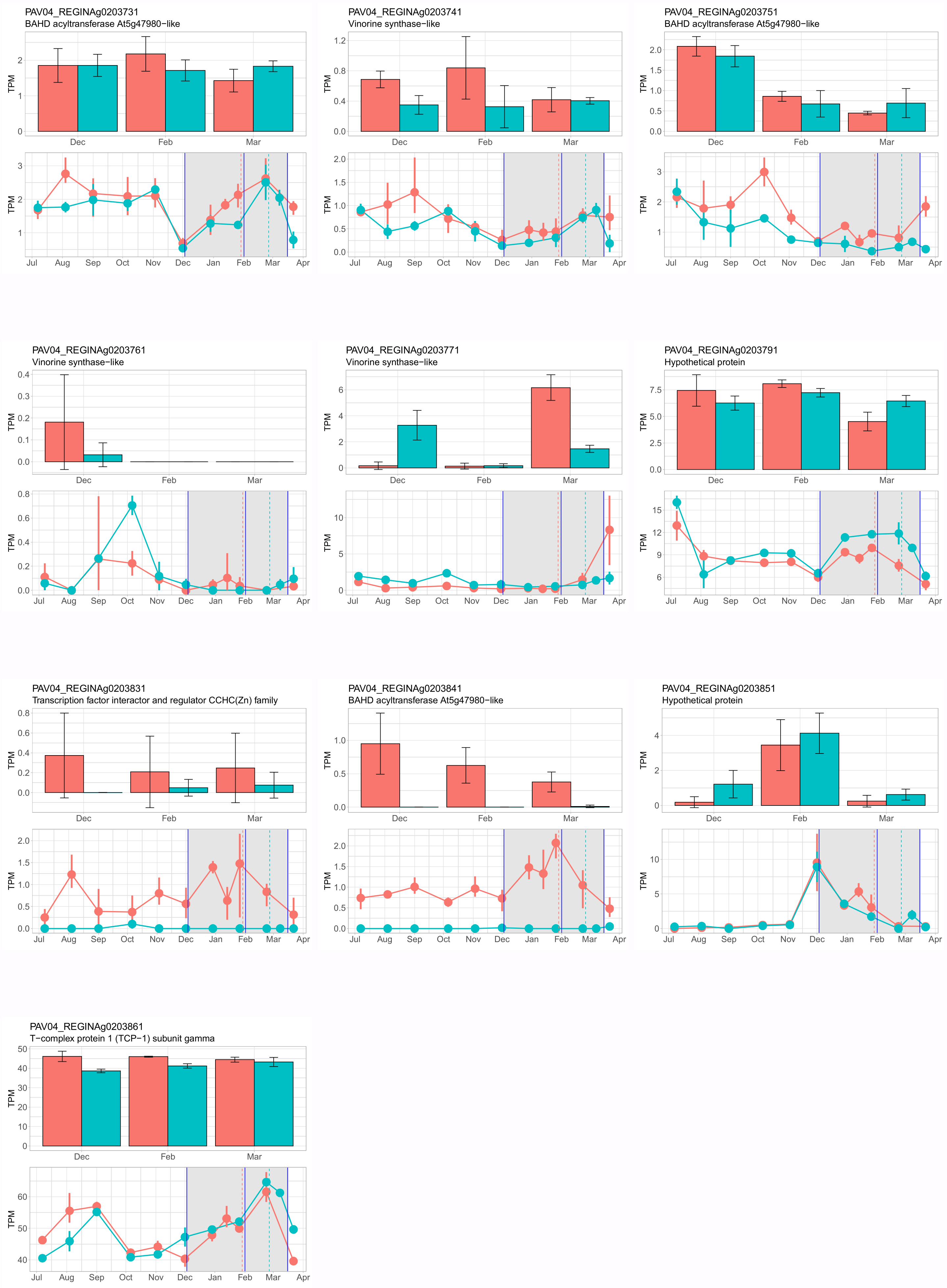

Fig. S4 Continued.

For each gene, two expression profiles are presented: the upper one is from the 2009/2010 RNA-seq analysis, across three sampling dates (3 December 2009, 1 February 2010 and 16 March 2010); the lower one is from the 2015/2016 RNA-seq analysis, between July 2015 and April 2016. Expression levels are measured in transcripts per million (TPM) in cultivars 'Regina' in blue and 'Garnet' in red. In the lower profile, the period colored in grey is the period in common with the first RNA-seq analysis, from December to March, and vertical blue lines correspond to the three sampling dates of the first analysis (3 December, 1 February and 16 March) (information added for potential qualitative comparison). Dotted lines correspond to the dormancy release dates for 'Garnet' (in red) and 'Regina' (in blue) in 2015/2016.
