# Supplementary material for: Plant N-acylethanolamines play a crucial role in defense and its variation in response to elevated CO2 and temperature in tomato
Source: Hortic Res. 2022 Oct 26;10(1):uhac242. doi: 10.1093/hr/uhac242 (PMC10108025; doi:10.1093/hr/uhac242)
Supplement: Web_Material_uhac242 [file web_material_uhac242.zip › Table. S1.pdf]

Table S1. Flowering date (FD) measurements (in Julian Days) in Populations #1 and #2.

Population	Year	FD stage	Min.	Mean	Max.	Std.dev.
Population #1	2008	BF	72.0	86.1	97.0	5.5
		FF	79.0	92.6	100.0	4.1
		EF	91.0	101.5	110.0	3.6
	2009	BF	77.0	88.8	96.0	3.6
		FF	85.0	93.4	100.0	3.3
		EF	99.0	104.6	110.0	2.2
	2010	BF	88.0	94.9	100.0	3.3
		FF	97.0	101.3	105.0	1.5
		EF	102.0	106.4	111.0	2.0
	2011	BF	79.0	85.5	92.0	2.5
		FF	85.0	90.7	95.0	2.3
		EF	91.0	96.0	100.0	2.5
	2012	BF	86.0	89.6	94.0	1.6
		FF	89.0	95.0	100.0	1.8
		EF	99.0	104.2	109.0	2.5
	2013	BF	85.0	96.8	101.0	3.5
		FF	98.0	102.7	105.0	1.6
		EF	100.0	108.3	113.0	2.4
	2014	BF	76.0	86.2	93.0	3.7
		FF	83.0	93.5	98.0	2.6
		EF	92.0	100.6	106.0	2.7
	2015	BF	91.0	99.8	103.0	2.1
		FF	100.0	104.4	108.0	1.7
		EF	106.0	111.6	118.0	2.5
	2016	BF	84.0	95.7	104.0	3.6
		FF	92.0	103.4	110.0	3.5
		EF	103.0	110.9	122.0	3.6
	2017	BF	70.0	82.5	89.0	3.7
		FF	76.0	88.5	96.0	2.5
		EF	89.0	97.3	104.0	3.0
Population #2	2018	BF	83.0	94.2	106.0	2.0
		FF	89.0	98.6	108.0	2.3
		EF	97.0	106.4	113.0	2.1
	2019	BF	75.0	83.4	91.0	2.4
		FF	81.0	90.3	102.0	3.1
		EF	91.0	101.8	113.0	3.6
	2021	BF	67.0	81.9	92.0	3.2
		FF	78.0	87.5	97.0	2.8
		EF	NA	NA	NA	NA

FD stages: BF, Beginning of flowering, FF, Full-flowering, EF, End of flowering; Min., Minimum; Max., Maximum; Std.dev., Standard deviation.
