# Supplementary material for: Plant N-acylethanolamines play a crucial role in defense and its variation in response to elevated CO2 and temperature in tomato
Source: Hortic Res. 2022 Oct 26;10(1):uhac242. doi: 10.1093/hr/uhac242 (PMC10108025; doi:10.1093/hr/uhac242)
Supplement: Web_Material_uhac242 [file web_material_uhac242.zip › Table. S10.pdf]

Table S10. Phenotypic and genomic data of the validation panels: the Regina × Lapins F_1 population (N = 115), accessions from the germplasm collection (N = 104) and the cultivars panel (N = 51).

**Regina × Lapins (N=115, planted in Toulenne)**																
ID	KASP_9.814	KASP_9.916	KASP_9.936	KASP_9.958	BF2006	BF2007	BF2008	BF2009	BF2010	BF2011	BF2012	BF2013	BF2014	BF2015	BF2016	**BFlsmeans**
RxL3	G:G	G:G	A:A	C:C	91	92	82	89	91	83	88	96	85	99	95	90.1
RxL7	G:G	G:G	A:A	C:C	91	95	84	90	94	85	88	98	87	98	96	91.5
RxL11	G:G	G:G	A:A	C:C	90	92	80	88	93	84	89	98	85	98	96	90.3
RxL13	G:G	G:G	A:A	C:C	93	95	82	89	93	85	88	98	87	98	96	91.3
RxL14	G:G	G:G	A:A	C:C	91	92	80	88	91	84	89	96	85	98	96	90.0
RxL15	G:G	G:G	A:A	C:C	91	95	82	88	91	85	89	99	87	98	97	91.1
RxL16	G:G	G:G	A:A	C:C	91	86	82	89	91	85	88	100	85	99	97	90.3
RxL27	G:G	G:G	A:A	C:C	91	95	84	89	92	85	89	98	86	99	94	91.1
RxL28	G:G	G:G	A:A	C:C	91	92	80	87	91	86	87	97	84	98	94	89.7
RxL29	G:G	G:G	A:A	C:C	91	95	84	87	95	86	89	97	88	100	97	91.7
RxL30	G:G	G:G	A:A	C:C	93	98	86	92	99	88	91	98	90	101	100	94.2
RxL36	G:G	G:G	A:A	C:C	91	86	78	84	89	81	87	91	83	95	91	86.9
RxL38	G:G	G:G	A:A	C:C	91	95	84	88	95	86	89	97	87	100	97	91.7
RxL40	G:G	G:G	A:A	C:C	91	95	84	89	96	86	88	98	85	99	97	91.6
RxL41	G:G	G:G	A:A	C:C	98	102	94	97	101	92	91	101	92	102	105	97.7
RxL42	G:G	G:G	A:A	C:C	91	95	82	89	96	87	89	96	89	100	97	91.9
RxL43	G:G	G:G	A:A	C:C	91	95	82	89	96	88	90	98	89	100	101	92.6
RxL48	G:G	G:G	A:A	C:C	96	98	91	89	96	86	89	98	87	99	97	93.3
RxL49	G:G	G:G	A:A	C:C	96	95	90	90	97	86	89	99	89	99	101	93.7
RxL51	G:G	G:G	A:A	C:C	91	95	90	89	95	85	89	99	86	99	97	92.3
RxL55	G:G	G:G	A:A	C:C	91	95	82	89	95	85	88	98	87	99	97	91.5
RxL57	G:G	G:G	A:A	C:C	91	95	82	89	95	87	90	96	86	100	96	91.5
RxL59	G:G	G:G	A:A	C:C	91	95	88	89	95	86	89	99	88	99	99	92.5
RxL60	G:G	G:G	A:A	C:C	91	99	84	90	96	86	89	99	88	100	100	92.9
RxL63	G:G	G:G	A:A	C:C	91	95	90	92	96	85	88	100	88	100	99	93.1
RxL66	G:G	G:G	A:A	C:C	91	100	88	91	97	86	90	100	90	100	99	93.8
RxL69	G:G	G:G	A:A	C:C	91	98	88	89	96	87	88	98	89	99	99	92.9
RxL71	G:G	G:G	A:A	C:C	91	95	84	89	96	86	89	98	89	97	98	92.0
RxL74	G:G	G:G	A:A	C:C	86	82	77	82	88	80	86	90	80	94	92	85.2
RxL75	G:G	G:G	A:A	C:C	96	99	91	92	98	87	90	100	90	100	102	95.0
RxL81	G:G	G:G	A:A	C:C	91	98	91	89	97	86	89	99	90	100	102	93.8
RxL82	G:G	G:G	A:A	C:C	91	95	88	89	96	86	89	99	87	98	97	92.3
RxL83	G:G	G:G	A:A	C:C	89	95	89	90	97	86	90	100	90	101	101	93.5

RxL85	G:G	G:G	A:A	C:C	91	92	82	89	96	85	89	98	86	98	95	91.0
RxL87	G:G	G:G	A:A	C:C	91	92	88	91	97	85	90	100	88	99	99	92.7
RxL88	G:G	G:G	A:A	C:C	91	98	92	92	99	86	90	100	90	100	102	94.5
RxL89	G:G	G:G	A:A	C:C	91	97	88	89	96	87	90	100	90	101	101	93.6
RxL94	G:G	G:G	A:A	C:C	91	99	91	91	98	86	91	99	91	101	102	94.5
RxL96	G:G	G:G	A:A	C:C	NA	99	93	90	96	82	90	100	90	100	NA	93.9
RxL97	G:G	G:G	A:A	C:C	91	92	82	86	91	84	86	96	83	97	92	89.1
RxL99	G:G	G:G	A:A	C:C	91	95	84	88	93	86	88	98	85	98	97	91.2
RxL100	G:G	G:G	A:A	C:C	91	98	84	87	94	85	89	100	87	99	99	92.1
RxL101	G:G	G:G	A:A	C:C	89	95	82	87	93	86	88	93	84	98	96	90.1
RxL106	G:G	G:G	A:A	C:C	91	99	88	89	97	86	89	100	89	99	99	93.3
RxL107	G:G	G:G	A:A	C:C	91	92	89	87	93	89	89	100	87	99	99	92.3
RxL111	G:G	G:G	A:A	C:C	91	98	84	89	97	87	90	100	89	100	99	93.1
RxL112	G:G	G:G	A:A	C:C	91	98	84	88	96	89	90	100	89	101	99	93.2
RxL114	G:G	G:G	A:A	C:C	91	92	82	86	93	85	87	97	86	99	95	90.3
RxL115	G:G	G:G	A:A	C:C	NA	95	91	87	96	87	89	100	87	100	100	93.1
RxL118	G:G	G:G	A:A	C:C	91	95	82	87	93	86	88	99	87	98	97	91.2
RxL123	G:G	G:G	A:A	C:C	91	95	84	82	91	83	87	101	83	97	92	89.6
RxL126	G:G	G:G	A:A	C:C	91	92	82	85	91	83	87	93	83	98	92	88.8
RxL129	G:G	G:G	A:A	C:C	91	95	82	89	95	86	89	98	85	99	93	91.1
RxL133	G:G	G:G	A:A	C:C	93	99	91	91	98	88	91	100	91	101	102	95.0
RxL10	A:G	A:G	A:A	T:C	91	99	88	95	101	89	89	101	92	103	104	95.6
RxL1	A:G	A:G	G:A	T:C	94	97	84	91	96	85	90	96	86	98	99	92.4
RxL2	A:G	A:G	G:A	T:C	96	102	91	94	100	91	94	100	90	103	101	96.5
RxL4	A:G	A:G	G:A	T:C	NA	97	88	92	94	87	93	98	87	100	96	93.1
RxL5	A:G	A:G	G:A	T:C	NA	99	94	96	101	92	96	102	93	103	104	97.9
RxL6	A:G	A:G	G:A	T:C	NA	102	88	93	99	88	92	100	91	100	104	95.6
RxL9	A:G	A:G	G:A	T:C	91	99	88	94	100	89	90	100	91	102	103	95.2
RxL17	A:G	A:G	G:A	T:C	91	100	91	93	100	89	92	100	91	99	104	95.5
RxL18	A:G	A:G	G:A	T:C	96	102	94	96	101	91	92	101	91	101	102	97.0
RxL19	A:G	A:G	G:A	T:C	91	98	88	92	98	89	92	99	90	101	100	94.4
RxL21	A:G	A:G	G:A	T:C	89	95	88	92	98	89	89	99	91	100	99	93.5
RxL22	A:G	A:G	G:A	T:C	96	99	91	92	98	88	92	99	90	101	102	95.3
RxL23	A:G	A:G	G:A	T:C	96	99	91	93	98	89	92	99	90	100	102	95.4
RxL25	A:G	A:G	G:A	T:C	98	104	94	96	101	91	92	100	95	103	106	98.2
RxL26	A:G	A:G	G:A	T:C	96	100	91	93	99	89	92	100	90	101	103	95.8
RxL31	A:G	A:G	G:A	T:C	93	99	86	91	98	88	89	97	88	98	97	93.1

RxL32	A:G	A:G	G:A	T:C	93	98	91	92	98	88	90	100	90	103	101	94.9
RxL33	A:G	A:G	G:A	T:C	91	99	82	87	95	86	88	98	87	99	95	91.5
RxL34	A:G	A:G	G:A	T:C	96	100	91	95	100	92	94	100	91	102	102	96.6
RxL35	A:G	A:G	G:A	T:C	91	98	91	91	98	86	90	99	89	100	99	93.8
RxL37	A:G	A:G	G:A	T:C	98	100	91	93	100	91	91	100	92	103	104	96.6
RxL39	A:G	A:G	G:A	T:C	96	99	91	92	98	92	94	100	91	96	102	95.5
RxL44	A:G	A:G	G:A	T:C	96	102	93	94	101	93	92	101	93	104	106	97.7
RxL45	A:G	A:G	G:A	T:C	96	101	91	94	100	89	89	100	90	103	102	95.9
RxL46	A:G	A:G	G:A	T:C	96	100	91	92	99	89	90	100	91	101	101	95.5
RxL47	A:G	A:G	G:A	T:C	96	99	91	92	100	89	91	100	93	103	102	96.0
RxL50	A:G	A:G	G:A	T:C	91	98	88	92	98	87	90	98	88	100	99	93.5
RxL53	A:G	A:G	G:A	T:C	98	101	91	93	99	88	94	99	86	101	102	95.6
RxL54	A:G	A:G	G:A	T:C	91	99	91	91	97	86	90	98	88	101	101	93.9
RxL58	A:G	A:G	G:A	T:C	91	95	84	90	96	85	89	99	87	99	96	91.9
RxL61	A:G	A:G	G:A	T:C	93	99	90	93	98	86	89	99	91	101	103	94.7
RxL62	A:G	A:G	G:A	T:C	96	98	91	93	98	89	90	99	NA	NA	102	95.3
RxL64	A:G	A:G	G:A	T:C	91	96	88	90	95	86	89	98	89	99	97	92.5
RxL65	A:G	A:G	G:A	T:C	89	82	77	85	89	82	87	92	81	98	95	87.0
RxL68	A:G	A:G	G:A	T:C	91	101	92	92	99	89	91	100	91	101	103	95.5
RxL70	A:G	A:G	G:A	T:C	91	95	92	92	98	87	91	99	87	99	99	93.6
RxL72	A:G	A:G	G:A	T:C	95	100	91	93	98	87	91	100	92	101	105	95.7
RxL73	A:G	A:G	G:A	T:C	95	99	88	92	97	87	91	101	88	100	99	94.3
RxL77	A:G	A:G	G:A	T:C	96	103	93	95	101	89	93	100	92	101	103	96.9
RxL78	A:G	A:G	G:A	T:C	89	95	82	88	94	85	89	93	81	98	94	89.8
RxL79	A:G	A:G	G:A	T:C	91	99	92	92	99	88	90	99	90	100	100	94.5
RxL84	A:G	A:G	G:A	T:C	NA	102	105	101	101	92	93	103	93	104	106	99.9
RxL90	A:G	A:G	G:A	T:C	96	100	91	92	99	87	91	100	91	101	97	95.0
RxL91	A:G	A:G	G:A	T:C	91	82	78	86	94	85	88	95	83	98	95	88.6
RxL92	A:G	A:G	G:A	T:C	96	100	96	96	100	89	93	101	93	102	106	97.5
RxL93	A:G	A:G	G:A	T:C	91	98	88	89	95	89	90	98	90	100	100	93.5
RxL95	A:G	A:G	G:A	T:C	91	100	89	91	97	87	90	99	88	100	100	93.8
RxL98	A:G	A:G	G:A	T:C	96	100	95	93	100	86	89	100	91	101	106	96.1
RxL102	A:G	A:G	G:A	T:C	91	100	88	89	97	92	89	99	91	100	99	94.1
RxL103	A:G	A:G	G:A	T:C	98	104	97	96	101	87	92	101	92	104	104	97.8
RxL104	G:G	A:G	G:A	T:C	91	100	92	92	99	89	91	101	90	100	100	95.0
RxL105	A:G	A:G	G:A	T:C	NA	NA	NA	92	99	86	89	100	93	103	105	95.5
RxL108	A:G	A:G	G:A	T:C	98	102	93	92	98	89	91	101	95	103	104	96.9

RxL109	A:G	A:G	G:A	T:C	96	101	92	93	98	87	91	101	91	102	104	96.0
RxL113	A:G	A:G	G:A	T:C	96	100	90	91	97	89	92	100	91	102	101	95.4
RxL116	A:G	A:G	G:A	T:C	96	100	92	92	99	89	91	101	91	101	104	96.0
RxL117	A:G	A:G	G:A	T:C	NA	102	96	95	101	91	91	101	92	103	104	97.5
RxL120	A:G	A:G	G:A	T:C	NA	103	94	93	100	90	91	101	92	102	104	96.9
RxL125	A:G	A:G	G:A	T:C	96	98	88	90	97	87	89	100	88	99	97	93.5
RxL130	A:G	A:G	G:A	T:C	91	99	90	91	97	87	90	100	88	100	102	94.1
RxL76	G:G	G:G	NA	C:C	89	92	82	87	94	84	87	93	86	98	95	89.7

Accessions from the Germplasm Collection (N=104, planted in Bourran)

Name	KASP_9.814	KASP_9.916	KASP_9.936	KASP_9.958	BF2014	BF2015	BF2016	BF2017	BF2018	BF2019	**BFlsmeans**
Angela	G:G	G:G	A:A	C:C	NA	98	103	NA	NA	NA	94.3
Arcina® Fercer	G:G	G:G	A:A	C:C	86	96	95	86	95	81	89.8
Bada	G:G	G:G	A:A	C:C	90	100	99	88	95	88	93.3
Bianca di Verona	G:G	G:G	A:A	C:C	88	95	91	79	90	80	87.2
Bigarreau de Juin	G:G	G:G	A:A	C:C	91	102	105	89	99	87	95.5
Bigarreau Hâtif Productif	G:G	G:G	A:A	C:C	92	103	NA	NA	99	87	95.1
Bigarreau Maria Gaucher	G:G	G:G	A:A	C:C	89	99	95	84	NA	86	91.3
Bigarreau Noire de Meched	G:G	G:G	A:A	C:C	92	101	102	90	NA	89	95.5
Bigarreau Saint Bruno	G:G	G:G	A:A	C:C	89	99	95	86	94	83	91.0
Bing	G:G	G:G	A:A	C:C	88	96	96	86	94	86	91.0
Blancale tardive	G:G	G:G	A:A	C:C	92	99	98	86	95	84	92.3
Cacouanne blanche	G:G	G:G	A:A	C:C	96	103	106	91	104	81	96.8
Caillou	G:G	G:G	A:A	C:C	85	92	87	79	89	82	85.7
Délice de Malicorne® Agoudel	G:G	G:G	A:A	C:C	90	97	91	83	91	81	88.8
Durona Prima di Vignola	G:G	G:G	A:A	C:C	NA	NA	98	89	99	84	93.6
Ferdouce	G:G	G:G	A:A	C:C	88	92	91	74	93	77	85.8
Ferlizac	G:G	G:G	A:A	C:C	90	100	91	83	95	81	90.0
Glacier	G:G	G:G	A:A	C:C	85	98	92	81	89	81	87.7
Grosse Rouge de la Faculté	G:G	G:G	A:A	C:C	88	100	91	86	94	84	90.5
Hartland	G:G	G:G	A:A	C:C	90	97	99	85	93	83	91.2
Jerusalem	G:G	G:G	A:A	C:C	96	102	105	89	104	91	97.8
Kassins Frühe	G:G	G:G	A:A	C:C	93	100	92	86	93	85	91.5
Kavics	G:G	G:G	A:A	C:C	90	100	102	89	101	88	95.0
Kordia	G:G	G:G	A:A	C:C	92	101	103	93	101	89	96.5
Kristin	G:G	G:G	A:A	C:C	91	100	98	88	96	87	93.3
Margit	G:G	G:G	A:A	C:C	90	98	102	87	95	86	93.0
Merton Crane	G:G	G:G	A:A	C:C	94	100	99	90	NA	88	94.9

O.T.E.A	G:G	G:G	A:A	C:C	91	98	NA	87	94	84	91.9
Olivette	G:G	G:G	A:A	C:C	91	96	94	84	92	82	89.8
Précoce d'Isigny	G:G	G:G	A:A	C:C	88	89	85	73	NA	74	82.5
Première	G:G	G:G	A:A	C:C	91	99	NA	NA	95	84	92.1
Rainier	G:G	G:G	A:A	C:C	89	95	96	83	95	83	90.2
Royalton	G:G	G:G	A:A	C:C	81	NA	94	82	90	82	87.2
Schmidt	G:G	G:G	A:A	C:C	88	98	96	87	93	83	90.8
Stark Hardy Giant	G:G	G:G	A:A	C:C	88	96	95	84	90	84	89.5
Sweet Ann	G:G	G:G	A:A	C:C	84	97	91	82	88	82	87.3
Sweetheart® Sumtare	G:G	G:G	A:A	C:C	86	101	92	80	90	77	87.7
V0255	G:G	G:G	A:A	C:C	88	97	92	79	92	82	88.3
V0566	G:G	G:G	A:A	C:C	91	99	102	88	96	86	93.7
Vega	G:G	G:G	A:A	C:C	85	98	91	82	91	78	87.5
Velvet	G:G	G:G	A:A	C:C	88	100	92	86	NA	81	90.1
Vernon	G:G	G:G	A:A	C:C	89	99	99	88	97	88	93.3
Vittoria	G:G	G:G	A:A	C:C	89	97	92	86	94	87	90.8
Xapata	G:G	G:G	A:A	C:C	88	96	91	74	93	82	87.3
4-84	G:G	G:G	A:A	C:C	90	97	91	86	91	83	89.7
Bigarreau Coeur	A:G	G:G	G:A	C:C	92	102	91	90	101	86	93.7
Gascogne double	A:G	NA	G:A	C:C	93	101	109	94	101	82	96.7
Primulat® Ferprime	A:G	G:G	G:A	C:C	81	101	91	74	88	77	85.3
Abouriou	G:G	G:G	G:A	T:C	80	96	89	74	90	78	84.5
Bigarreau Camus de Vénasque	G:G	G:G	G:A	T:C	93	100	102	92	NA	88	95.7
Bigarreau Hâtif Burlat	G:G	G:G	G:A	T:C	89	95	95	83	93	81	89.3
Bigarreau Pelissier	G:G	G:G	G:A	T:C	91	100	102	89	101	87	95.0
Blancale précoce	A:G	A:G	G:A	T:C	84	94	90	74	90	74	84.3
Bruelles	A:G	A:G	G:A	T:C	82	95	89	79	88	82	85.8
Cerna	G:G	G:G	G:A	T:C	88	97	96	83	94	82	90.0
Durone Nero Seconda di Vignola	G:G	G:G	G:A	T:C	92	103	105	88	101	88	96.2
Duroni 3	G:G	G:G	G:A	T:C	92	101	105	89	103	88	96.3
Early Rivers	G:G	G:G	G:A	T:C	88	96	91	83	90	81	88.2
Etienne Thuilleaux	G:G	G:G	G:A	T:C	82	94	87	79	91	82	85.8
Ferdiva	G:G	G:G	G:A	T:C	94	103	102	87	99	86	95.2
Fertard	G:G	G:G	G:A	T:C	92	101	106	93	101	87	96.7
Franc Tellier	G:G	G:G	G:A	T:C	81	94	107	NA	NA	80	89.8
Gascogne tardive de Seninghem	G:G	G:G	G:A	T:C	90	102	105	90	103	88	96.3
Giorgia	G:G	G:G	G:A	T:C	92	100	102	87	93	82	92.7

Graffioni	G:G	G:G	G:A	T:C	95	103	106	93	99	91	97.8
Grosse blanche de Verchocq	G:G	G:G	G:A	T:C	90	100	99	88	99	83	93.2
Grosse Schwarze Knorpelkirsche	G:G	G:G	G:A	T:C	83	97	90	76	89	80	85.8
Guigne Hâtive d'Annonay	G:G	G:G	G:A	T:C	86	97	97	88	91	83	90.3
Impériale	G:G	G:G	G:A	T:C	91	100	102	88	95	84	93.3
Katalin	G:G	G:G	G:A	T:C	91	99	103	87	95	NA	93.5
La Chalonnaise (n°2)	G:G	G:G	G:A	T:C	86	99	93	81	91	81	88.5
Lambert	G:G	G:G	G:A	T:C	89	101	NA	NA	NA	86	93.0
Larian	G:G	G:G	G:A	T:C	88	101	104	88	95	86	93.7
Le cardan	G:G	G:G	G:A	T:C	97	103	109	94	104	91	99.7
Linda	G:G	G:G	G:A	T:C	91	101	106	90	98	88	95.7
Merveille de Saint Genis Laval	G:G	G:G	G:A	T:C	92	99	96	90	92	84	92.2
Nanyo	G:G	G:G	G:A	T:C	97	103	112	99	106	NA	101.9
Ohio Beauty	G:G	G:G	G:A	T:C	90	98	94	86	95	84	91.2
Rippert Botond	G:G	G:G	G:A	T:C	96	103	105	90	99	88	96.8
Rubin	G:G	G:G	G:A	T:C	92	100	106	90	99	NA	95.9
Sandra rose	G:G	G:G	G:A	T:C	91	99	105	89	99	84	94.5
Satonishiki	G:G	G:G	G:A	T:C	92	100	95	86	99	87	93.2
Skeena	G:G	G:G	G:A	T:C	91	100	103	88	97	87	94.3
Stella	G:G	G:G	G:A	T:C	90	99	96	87	91	83	91.0
Targonnais	G:G	G:G	G:A	T:C	92	99	99	88	99	87	94.0
Turca	G:G	G:G	G:A	T:C	93	100	105	90	91	89	94.7
Uriase de Bistrita	G:G	G:G	G:A	T:C	93	100	102	88	96	88	94.5
V4135	G:G	G:G	G:A	T:C	89	97	98	83	95	83	90.8
Versaillaise	G:G	G:G	G:A	T:C	94	102	103	94	103	86	97.0
Vista	G:G	G:G	G:A	T:C	84	99	89	74	89	81	86.0
7616-4	G:G	G:G	G:A	T:C	91	101	99	86	96	86	93.2
Bigarreau Courte Queue	A:G	G:G	G:G	T:C	82	93	89	74	87	77	83.7
Bigarreau Grand	A:G	G:G	G:G	T:C	91	96	98	89	99	87	93.3
Bigarreau Guillaume	A:G	G:G	G:G	T:C	92	101	104	93	103	NA	97.1
Cyprès	A:G	G:G	G:G	T:C	89	99	96	88	94	86	92.0
Précoce Bernard	A:G	G:G	G:G	T:C	88	94	91	81	93	82	88.2
Valerij Chkalov	A:G	G:G	G:G	T:C	83	92	91	76	90	77	84.8
Bigarreau de Mai	G:G	G:G	G:G	T:T	88	95	95	83	93	83	89.5
Bigarreau Noir d'Ecully	G:G	G:G	G:G	T:T	89	96	NA	NA	91	81	89.1
Blanchère	G:G	G:G	G:G	T:T	88	95	93	NA	NA	83	89.1
Géant d'Hedelfingen	G:G	G:G	G:G	T:T	95	103	95	NA	99	91	95.4

Scwecja A	G:G	G:G	G:G	T:T	84	95	91	82	95	81	88.0
V3858	A:G	A:G	G:G	T:T	87	95	90	83	90	83	88.0
Yuko Nishiki	G:G	G:G	G:G	T:T	82	97	92	86	93	84	89.0
**Cultivars (N=51, planted in Toulenne)**											
Name	KASP_9.814	KASP_9.916	KASP_9.936	KASP_9.958	BF2014	BF2015	BF2016	BF2017	BF2018	BF2019	**BFlsmeans**
Arcina® Fercer	G:G	G:G	A:A	C:C	81	97	86	76	88	79	84.5
Bigarreau-Marmotte	G:G	G:G	A:A	C:C	77	98	86	73	87	77	83.0
Canada Giant®	G:G	G:G	A:A	C:C	90	101	97	88	95	84	92.5
Délice de Malicorne® Agoudel	G:G	G:G	A:A	C:C	77	97	87	83	88	81	85.5
Earlired	G:G	G:G	A:A	C:C	81	98	90	78	90	82	86.5
Ferdouce	G:G	G:G	A:A	C:C	83	98	87	79	83	81	85.2
Feria	G:G	G:G	A:A	C:C	87	98	88	80	89	79	86.8
Fermina	G:G	G:G	A:A	C:C	83	99	92	81	91	83	88.2
Fertille	G:G	G:G	A:A	C:C	84	99	91	81	88	83	87.7
Giantred	G:G	G:G	A:A	C:C	84	99	89	78	92	NA	87.1
Kordia	G:G	G:G	A:A	C:C	84	101	91	84	91	83	89.0
Lapins	G:G	G:G	A:A	C:C	84	99	93	83	91	83	88.8
Merveille des Préaux	G:G	G:G	A:A	C:C	89	102	93	88	93	85	91.7
SF91131	G:G	G:G	A:A	C:C	89	100	92	80	91	82	89.0
Staccato®	G:G	G:G	A:A	C:C	NA	NA	NA	85	96	82	91.3
Stark Hardy Giant	G:G	G:G	A:A	C:C	83	99	90	79	91	80	87.0
Sumcoja	G:G	G:G	A:A	C:C	80	96	89	79	87	74	84.2
Sweet Early® Parano 1	G:G	G:G	A:A	C:C	76	94	86	76	82	70	80.7
Van	G:G	G:G	A:A	C:C	80	96	89	78	88	81	85.3
Ferpin	A:G	G:G	G:A	C:C	87	97	87	84	89	82	87.7
Primulat® Ferprime	A:G	G:G	G:A	C:C	72	93	83	71	71	72	77.0
Badacsonyi	G:G	G:G	G:A	T:C	NA	NA	NA	88	96	85	93.3
Belle de Fabrega	G:G	G:G	G:A	T:C	86	99	93	80	90	82	88.3
Bellise® Bedel	G:G	G:G	G:A	T:C	75	94	85	77	83	76	81.7
Bigarreau Camus de Vénasque	G:G	G:G	G:A	T:C	86	100	92	83	91	84	89.3
Bigarreau Hâtif Burlat	G:G	G:G	G:A	T:C	76	96	84	78	83	75	82.0
Black Star	G:G	G:G	G:A	T:C	77	95	84	78	83	84	83.5
C-73-5	G:G	G:G	G:A	T:C	92	104	98	90	99	89	95.3
Durona di Vignola II	G:G	G:G	G:A	T:C	83	100	91	83	89	82	88.0
Duroni 3	G:G	G:G	G:A	T:C	93	100	97	86	96	86	93.0
Ferdiva	G:G	G:G	G:A	T:C	93	102	103	88	97	88	95.2
Fernier	G:G	G:G	G:A	T:C	86	99	94	85	93	83	90.0

Ferobri	G:G	G:G	G:A	T:C	78	98	85	77	82	77	82.8
Ferpact	G:G	G:G	G:A	T:C	87	98	88	80	91	82	87.7
Fertard	G:G	G:G	G:A	T:C	90	102	94	85	94	84	91.5
Folfer	G:G	G:G	G:A	T:C	73	92	83	70	74	68	76.7
Girodel	G:G	G:G	G:A	T:C	84	98	88	81	87	NA	86.3
Grace Star	G:G	G:G	G:A	T:C	80	98	87	80	90	80	85.8
Napoleon	G:G	G:G	G:A	T:C	83	98	89	77	87	77	85.2
Penny	G:G	G:G	G:A	T:C	95	104	105	90	100	87	96.8
Rubin	G:G	G:G	G:A	T:C	90	102	97	85	92	85	91.8
Sandar	G:G	G:G	G:A	T:C	90	102	94	84	92	82	90.7
Stella	G:G	G:G	G:A	T:C	NA	NA	NA	86	92	83	90.7
Sumbigo	G:G	G:G	G:A	T:C	78	94	89	72	NA	NA	82.1
Sunburst	G:G	G:G	G:A	T:C	90	100	96	83	95	84	91.3
Techlovan	G:G	G:G	G:A	T:C	87	100	95	NA	93	83	90.3
Vanda	G:G	G:G	G:A	T:C	92	102	100	88	98	89	94.8
Viva	G:G	G:G	G:A	T:C	NA	NA	NA	83	92	83	89.7
4L-15 G1 V2220	G:G	G:G	G:A	T:C	90	101	95	88	94	82	91.7
Géant d'Hedelfingen	G:G	G:G	G:G	T:T	83	102	92	84	92	84	89.5
Tieton	G:G	G:G	G:G	T:T	84	99	92	NA	NA	83	88.4

BF, beginning of flowering scored in Julian Days (JDs); NA, missing data
