# Supplementary material for: Plant N-acylethanolamines play a crucial role in defense and its variation in response to elevated CO2 and temperature in tomato
Source: Hortic Res. 2022 Oct 26;10(1):uhac242. doi: 10.1093/hr/uhac242 (PMC10108025; doi:10.1093/hr/uhac242)
Supplement: Web_Material_uhac242 [file web_material_uhac242.zip › Table. S2.pdf]

Table S2. Temperatures across the 13 years of flowering date (FD) evaluation (period from October to April).

Temperature	Season	October	November	December	January	February	March	April
Minimum monthly temperature (°C)	2007/2008	7.3	1.3	1.6	3.8	3.1	5.5	6.5
	2008/2009	7.2	5.3	1.6	1.2	1.6	2.9	6.8
	2009/2010	8.3	8.3	2.9	0.1	1.6	3.6	6.4
	2010/2011	7.9	6.4	0.8	1.4	3.6	4.4	7.3
	2011/2012	7.7	8.7	5.5	3.6	-3.4	2.0	6.3
	2012/2013	9.9	5.9	4.3	2.9	1.5	4.6	6.5
	2013/2014	10.8	5.5	1.3	5.5	4.9	4.0	7.6
	2014/2015	10.3	8.9	3.0	2.3	1.6	5.4	7.0
	2015/2016	7.3	7.7	4.9	5.5	4.7	4.2	6.4
	2016/2017	7.2	5.4	1.9	-1.2	4.1	6.1	4.2
	2017/2018	9.4	2.1	2.6	6.6	0.5	5.0	8.8
	2018/2019	7.9	5.2	5.7	1.3	2.7	4.3	6.3
	2020/2021	9.1	5.2	4.0	2.3	7.4	4.0	3.8
Mean monthly temperature (°C)	2007/2008	13.0	6.8	5.6	7.8	9.2	9.3	11.8
	2008/2009	13.2	8.7	5.1	4.8	6.7	9.2	11.9
	2009/2010	14.5	11.9	6.6	3.5	5.8	9.0	13.0
	2010/2011	13.2	9.6	4.6	5.2	8.2	10.0	14.6
	2011/2012	14.5	12.5	9.1	6.6	1.7	9.9	10.6
	2012/2013	14.6	9.9	8.2	6.0	5.7	9.8	11.9
	2013/2014	15.2	8.7	5.3	8.6	8.5	9.8	13.4
	2014/2015	15.7	12.3	6.1	5.5	5.6	9.6	13.3
	2015/2016	12.5	11.5	9.0	8.4	8.1	9.0	11.5
	2016/2017	12.1	9.4	5.6	3.0	8.5	11.0	12.1
	2017/2018	14.7	7.2	6.4	9.1	4.4	9.2	13.8
	2018/2019	13.4	9.3	8.6	5.0	8.3	10.5	12.0
	2020/2021	13.0	10.1	7.7	5.4	11.6	9.8	10.9
Maximum monthly temperature (°C)	2007/2008	18.7	12.3	9.6	11.8	15.3	13.0	17.2
	2008/2009	19.1	12.2	8.7	8.4	11.8	15.5	17.1
	2009/2010	20.7	15.4	10.3	6.9	10.1	14.4	19.7
	2010/2011	18.5	12.8	8.4	9.1	12.7	15.7	21.9
	2011/2012	21.3	16.4	12.7	9.6	6.8	17.8	15.0
	2012/2013	19.4	13.9	12.1	9.2	9.9	14.9	17.5
	2013/2014	21.6	12.6	10.6	13.1	13.2	16.6	19.9
	2014/2015	23.4	16.9	9.9	9.8	10.6	14.7	20.1
	2015/2016	19.0	16.8	14.5	12.4	12.7	14.4	17.3
	2016/2017	19.1	14.1	11.1	8.6	14.3	17.0	19.9
	2017/2018	22.2	13.9	10.5	12.1	9.2	14.1	19.6
	2018/2019	20.5	14.9	12.2	9.2	15.4	17.1	18.0
	2020/2021	18.6	17.3	11.6	9.7	16.9	16.3	17.9
