# Supplementary material for: Plant N-acylethanolamines play a crucial role in defense and its variation in response to elevated CO2 and temperature in tomato
Source: Hortic Res. 2022 Oct 26;10(1):uhac242. doi: 10.1093/hr/uhac242 (PMC10108025; doi:10.1093/hr/uhac242)
Supplement: Web_Material_uhac242 [file web_material_uhac242.zip › Table. S3.pdf]

Table S3. Spearman correlation coefficients between FD stages (BF, beginning of flowering; FF, full flowering and EF, end of flowering) across years in Populations #1 and #2.

**Population#1**	BF2008	FF2008	EF2008	BF2009	FF2009	EF2009	BF2010	FF2010	EF2010	BF2011	FF2011	EF2011	BF2012	FF2012	EF2012	BF2013	FF2013	EF2013	BF2014	FF2014	EF2014	BF2015	FF2015	EF2015	BF2016	FF2016	EF2016	BF2017	FF2017
BF2008																													
FF2008	0.91*																												
EF2008	0.75*	0.85*																											
BF2009	0.84*	0.84*	0.74*																										
FF2009	0.82*	0.82*	0.73*	0.97*																									
EF2009	0.73*	0.75*	0.75*	0.85*	0.89*																								
BF2010	0.81*	0.82*	0.73*	0.95*	0.95*	0.85*																							
FF2010	0.77*	0.77*	0.72*	0.89*	0.87*	0.78*	0.90*																						
EF2010	0.71*	0.77*	0.77*	0.81*	0.79*	0.80*	0.84*	0.82*																					
BF2011	0.72*	0.74*	0.69*	0.86*	0.86*	0.73*	0.90*	0.81*	0.76*																				
FF2011	0.72*	0.73*	0.68*	0.83*	0.84*	0.73*	0.87*	0.76*	0.76*	0.93*																			
EF2011	0.73*	0.70*	0.66*	0.82*	0.84*	0.79*	0.81*	0.72*	0.76*	0.79*	0.86*																		
BF2012	0.76*	0.73*	0.69*	0.84*	0.83*	0.74*	0.86*	0.80*	0.77*	0.81*	0.82*	0.80*																	
FF2012	0.70*	0.73*	0.67*	0.80*	0.79*	0.69*	0.83*	0.77*	0.70*	0.79*	0.79*	0.71*	0.85*																
EF2012	0.59*	0.64*	0.64*	0.68*	0.69*	0.73*	0.74*	0.64*	0.75*	0.65*	0.68*	0.69*	0.71*	0.76*															
BF2013	0.77*	0.82*	0.76*	0.86*	0.83*	0.75*	0.88*	0.82*	0.80*	0.86*	0.86*	0.78*	0.81*	0.81*	0.72*														
FF2013	0.71*	0.76*	0.71*	0.80*	0.79*	0.73*	0.84*	0.76*	0.75*	0.80*	0.80*	0.71*	0.78*	0.82*	0.75*	0.92*													
EF2013	0.73*	0.77*	0.74*	0.80*	0.81*	0.79*	0.85*	0.77*	0.78*	0.80*	0.80*	0.73*	0.80*	0.85*	0.79*	0.89*	0.92*												
BF2014	0.78*	0.76*	0.71*	0.84*	0.85*	0.75*	0.86*	0.80*	0.75*	0.85*	0.81*	0.78*	0.86*	0.79*	0.66*	0.84*	0.79*	0.82*											
FF2014	0.79*	0.71*	0.66*	0.80*	0.82*	0.74*	0.81*	0.75*	0.73*	0.81*	0.78*	0.82*	0.82*	0.70*	0.64*	0.76*	0.70*	0.75*	0.91*										
EF2014	0.70*	0.71*	0.72*	0.75*	0.75*	0.77*	0.77*	0.75*	0.81*	0.73*	0.70*	0.76*	0.78*	0.70*	0.74*	0.78*	0.73*	0.80*	0.85*	0.85*									
BF2015	0.78*	0.77*	0.74*	0.86*	0.87*	0.81*	0.88*	0.81*	0.81*	0.85*	0.84*	0.84*	0.87*	0.82*	0.75*	0.85*	0.81*	0.85*	0.91*	0.88*	0.86*								
FF2015	0.79*	0.74*	0.71*	0.83*	0.84*	0.80*	0.84*	0.79*	0.78*	0.79*	0.77*	0.80*	0.86*	0.76*	0.73*	0.77*	0.72*	0.79*	0.89*	0.88*	0.88*	0.92*							
EF2015	0.70*	0.67*	0.64*	0.74*	0.75*	0.76*	0.77*	0.73*	0.79*	0.68*	0.71*	0.76*	0.78*	0.67*	0.75*	0.72*	0.68*	0.74*	0.77*	0.80*	0.86*	0.86*	0.90*						
BF2016	0.74*	0.73*	0.68*	0.79*	0.78*	0.71*	0.78*	0.74*	0.71*	0.75*	0.73*	0.75*	0.78*	0.72*	0.60*	0.78*	0.72*	0.73*	0.86*	0.83*	0.76*	0.85*	0.80*	0.69*					
FF2016	0.78*	0.77*	0.73*	0.82*	0.83*	0.77*	0.84*	0.78*	0.76*	0.77*	0.76*	0.80*	0.83*	0.77*	0.69*	0.82*	0.79*	0.82*	0.89*	0.87*	0.83*	0.88*	0.86*	0.78*	0.93*				
EF2016	0.71*	0.72*	0.72*	0.77*	0.78*	0.76*	0.82*	0.77*	0.78*	0.74*	0.72*	0.75*	0.81*	0.75*	0.73*	0.80*	0.79*	0.84*	0.86*	0.83*	0.87*	0.86*	0.85*	0.81*	0.88*	0.95*			
BF2017	0.79*	0.79*	0.73*	0.88*	0.88*	0.80*	0.88*	0.83*	0.80*	0.84*	0.81*	0.81*	0.86*	0.81*	0.69*	0.88*	0.83*	0.84*	0.93*	0.88*	0.85*	0.91*	0.87*	0.78*	0.85*	0.89*	0.86*		
FF2017	0.78*	0.79*	0.75*	0.87*	0.87*	0.80*	0.87*	0.81*	0.81*	0.83*	0.81*	0.81*	0.85*	0.81*	0.75*	0.87*	0.83*	0.85*	0.91*	0.87*	0.86*	0.92*	0.88*	0.82*	0.83*	0.89*	0.88*	0.96*	
EF2017	0.71*	0.75*	0.70*	0.82*	0.82*	0.79*	0.82*	0.76*	0.83*	0.77*	0.76*	0.76*	0.82*	0.76*	0.80*	0.83*	0.78*	0.82*	0.84*	0.81*	0.88*	0.88*	0.88*	0.88*	0.80*	0.85*	0.88*	0.90*	0.93*

**Population#2**	BF2018	FF2018	EF2018	BF2019	FF2019	EF2019	BF2021
BF2018							
FF2018	0.80*						
EF2018	0.75*	0.76*					
BF2019	0.78*	0.71*	0.69*				
FF2019	0.73*	0.71*	0.69*	0.90*			
EF2019	0.69*	0.67*	0.71*	0.80*	0.87*		
BF2021	0.76*	0.69*	0.66*	0.82*	0.76*	0.72*	
FF2021	0.74*	0.68*	0.68*	0.81*	0.78*	0.74*	0.94*

significant correlation: *, p < 0,001
