# Supplementary material for: Plant N-acylethanolamines play a crucial role in defense and its variation in response to elevated CO2 and temperature in tomato
Source: Hortic Res. 2022 Oct 26;10(1):uhac242. doi: 10.1093/hr/uhac242 (PMC10108025; doi:10.1093/hr/uhac242)
Supplement: Web_Material_uhac242 [file web_material_uhac242.zip › Table. S4.pdf]

Table S4. Origin, position on the 'Regina' genome, sequence and primers sequences of the 17 KASP markers used for the fine mapping of the QTL on LG4 of 'Regina'.

KASP	SNP origin	Position on 'Regina' genome sequence (bp)	Genotype 'Regina'	Genotype 'Garnet'	Marker sequence	AlleleX	AlleleY	Primer_AlleleX	Primer_AlleleY	Primer_Common
KASP_9.269	RNA-seq (IGV software)	9268774	C/T	T/T	ATTAAATATCAGCTTCCTGGAGAGGATCTTGATGCCTTAGTTTCTGTATCTTGCGACGAGGATYTGCAGAATATGATGGAAGAATGGAATGAACTAGAAGATAAAGAAGGACCACAAAAGCTTAGG	C	T	GAAGGTGACCAAGTTCATGCTATTCCATTCTTCCATCATATTCTGCAG	GAAGGTCGGAGTCAACGGATTCATTCCATTCTTCCATCATATTCTGCAA	TTAGTTTCTGTATCTTGCGACGAGGAT
KASP_9.271	GBS	9270618	G/A	A/A	TGAATGTCTTCCGCAAATTTAGGCTGAGAATCCACATGGACCATTGAAACTCCAGCTGCTATTAATGGTGTGAGATGATAAGATCTACTRTCTCCTTCCACATCATTTCCAATCACAGACGGAAAACCAAGATCCTGGTCAATAAGAGAAACATCTTTTTTATCAAACCCTT	A	G	GAAGGTGACCAAGTTCATGCTGATTGGAAATGATGTGGAAGGAGAT	GAAGGTCGGAGTCAACGGATTGGAAATGATGTGGAAGGAGAC	CAGCTGCTATTAATGGTGTGAGATGATAA
KASP_9.456	RNA-seq (IGV software)	9455997	A/T	A/A	ATTTAAACACTTTACGAAGAAGTGTCGATGGGTGTGTCGGACTGCGCTCCCCATTCWGTCCAAGATCCATCATAGACTGCTACTTCAGGCTTACCGAGTCGATGAAGACCCTGGATGA	A	T	GAAGGTGACCAAGTTCATGCTAGCAGTCTATGATGGATCTTGGACT	GAAGGTCGGAGTCAACGGATTAGCAGTCTATGATGGATCTTGGACA	TGTCGGACTGCGCTCCCCATT
KASP_9.727	GBS	9726870	A/G	A/A	CCAAACACCAGAGTTTGCGTATGAAAATTGGACACCCAATTGAGGATTGATGATAATAAATTTGGTATTATRAAAATTGACAAATAACCATATAAATATATTGTATGGCTATGGAGCTGCAACATATCCTAGTTAACTTGAAACTTAATTTGATAGCTTAGGGGCCAA	A	G	GAAGGTGACCAAGTTCATGCTTGAGGATTGATGATAATAAATTTGGTATTATA	GAAGGTCGGAGTCAACGGATTGAGGATTGATGATAATAAATTTGGTATTATG	ATGTTGCAGCTCCATAGCCATACAATATA
KASP_9.780	GBS	9780346	A/G	A/A	ACCATATGATCAGCTGGTAGCATATGGTAAGTACATTAGACCCATAGCCTCTTGCAGCCAGTAGTCGTCCTCRTCATATTTCATCATCTGTAAATTAAACGATGTACATGATTCAATAATGTCAAAATGATTTCATAATAAGTAGCTAGATGATTGTACTTT	A	G	GAAGGTGACCAAGTTCATGCTACATCGTTTAATTTACAGATGATGAAATATGAT	GAAGGTCGGAGTCAACGGATTCATCGTTTAATTTACAGATGATGAAATATGAC	CCCATAGCCTCTTGCAGCCAGTA
KASP_9.781	RNA-seq (IGV software)	9780552	C/G	C/C	CGCTTGTATTGTACAAGTATATGATCTAACAAGCTAGCTAATTAGTCTTTT[C/G]TACTCGTAAAATTAATTGGTGCATTGGTTTTAATGCGTATCTACGTATACG	C	G	GAAGGTGACCAAGTTCATGCTCCAATGCACCAATTAATTTTACGAGTAG	GAAGGTCGGAGTCAACGGATTCCAATGCACCAATTAATTTTACGAGTAC	GTACAAGTATATGATCTAACAAGCTAGCTA
KASP_9.801	RNA-seq (IGV software)	9800899	A/G	A/A	GAAAGTCTTGATTGCAAAGCTTGGAGTGACACTAGAAGACTCAAAAATTGAT[A/G]CGCTCATTGGCTTGAGGATGGTTAACATGAATTTCTACCCAACATGCCCTGA	A	G	GAAGGTGACCAAGTTCATGCTCCATCCTCAAGCCAATGAGCGT	GAAGGTCGGAGTCAACGGATTCATCCTCAAGCCAATGAGCGC	GCTTGGAGTGACACTAGAAGACTCAA
KASP_9.814	RNA-seq (IGV software)	9813748	G/A	G/G	TAAGTACTTTTATTGACCTCGACAAGGGAATATTGTTTGTAGAGTCACCAC[G/A]ATGCCAAGTGAGACTGCCAATCAACTTTATCACAGATTCATGTAATGGTGG	G	A	GAAGGTGACCAAGTTCATGCTGGAATATTGTTTGTAGAGTCACCACG	GAAGGTCGGAGTCAACGGATTGGAATATTGTTTGTAGAGTCACCACA	GTTGATTGGCAGTCTCACTTGGCAT
KASP_9.890	RNA-seq (IGV software)	9889761	A/C	C/C	AGGGAGGGCTTATGTTGTTGTTAATGGAGTCTCTGGACCGCTTTCTTGGTAGACTAATCGGAAMGTTGTAGGTGAGGCCGCTATCGGATTTCATGGCTGCGGTGGTTGAGGTCTTCGGAGGCATTA	A	C	GAAGGTGACCAAGTTCATGCTGATAGCGGCCTCACCTACAACT	GAAGGTCGGAGTCAACGGATTATAGCGGCCTCACCTACAACG	CCGCTTTCTTGGTAGACTAATCGGAA
KASP_9.916	RNA-seq (IGV software)	9915521	G/A	G/G	GTTTCAAGATTCTTAACGTGGTCAATTACATATACAAGCCTCGGGCCTTTCC[G/A]AAGCCCTGAGGAAACAGAAGCACTCCTTAGAACCATAACTAGAATGTAAAGT	G	A	GAAGGTGACCAAGTTCATGCTCAAGCCTCGGGCCTTTCCG	GAAGGTCGGAGTCAACGGATTATACAAGCCTCGGGCCTTTCCA	AGTGCTTCTGTTTCCTCAGGGCTT
KASP_9.933	RNA-seq (IGV software)	9932706	C/T	C/C	ACCTGAATCTACCAACAGACTTCACAGGAAGTCGAGAGAGTATGTCTGCAA[C/T]GATATCTTCTGAAAGGTTTGCCATTTACGCCTAAAAAGTTCACAACTTGAC	C	T	GAAGGTGACCAAGTTCATGCTAAATGGCAAACCTTTCAGAAGATATCG	GAAGGTCGGAGTCAACGGATTGTAAATGGCAAACCTTTCAGAAGATATCA	GGAAGTCGAGAGAGTATGTCTGCAA
KASP_9.935	RNA-seq (IGV software)	9935374	A/G	G/G	AAACAAAGACACCCTCTCAAAACACCAGTTGTAAATCTCTTATAATCTGTGACAGATTCAGGCRGGGGCAAATCAGAGAACTTCTCCTGTGCTAAATCAAAAGCAGCAATCACACAGGTACCTCCA	A	G	GAAGGTGACCAAGTTCATGCTCTTATAATCTGTGACAGATTCAGGCA	GAAGGTCGGAGTCAACGGATTCTTATAATCTGTGACAGATTCAGGCG	GATTTAGCACAGGAGAAGTTCTCTGATTT
KASP_9.936	RNA-seq (IGV software)	9935681	A/G	A/A	ATGCATGATGAAACATAGCTTCTTGTGGTTCTGGAGGACAAATATGACTTG[A/G]CATGGGGCAATCTGGTACTCTCAAGGACTCTCTAGTGGTAGGATTGAATAT	A	G	GAAGGTGACCAAGTTCATGCTGAGAGTACCAGATTGCCCCATGT	GAAGGTCGGAGTCAACGGATTAGAGTACCAGATTGCCCCATGC	TTGTGGTTCTGGAGGACAAATATGACTT
KASP_9.958	RNA-seq (IGV software)	9957746	C/T	C/C	TGCTCGGTATGGGTGATCCTCGAGATCAAAGGCCAAAATCAGCTCAGAAAGT[C/T]AGACTAAATATGCGTTTTTCTCAATATGGCTTTGATACAGGTAGCCATAGGA	C	T	GAAGGTGACCAAGTTCATGCTAAGGCCAAAATCAGCTCAGAAAGTC	GAAGGTCGGAGTCAACGGATTAAAGGCCAAAATCAGCTCAGAAAGTT	GCTACCTGTATCAAAGCCATATTGAGAAA
KASP_9.970	RNA-seq (IGV software)	9970195	A/T	A/A	TTCGATAGAGAGGTGGTAATGTTGAGGAGGAGACATGCAGAGAAGGTGAGC[A/T]TGTTCTATGGAACGATCAGAGAGCAATATCTGAAGAATAAGGGCTATTGGA	A	T	GAAGGTGACCAAGTTCATGCTCTCTGATCGTTCCATAGAACAT	GAAGGTCGGAGTCAACGGATTGCTCTCTGATCGTTCCATAGAACAA	TGTTGAGGAGGAGACATGCAGAGAA
KASP_10.090	GBS	10089579	A/G	A/A	CGTCGGAGCGAGGCGGCAGCAAATCGCCTCAAGGCATCGTTTGTTTCRCGACTTGCACGTTTAGCACGGGGAAGGCGTGAAGAGGCGTTTCAAGGCGCAGCGAGGTGGGGC	A	G	GAAGGTGACCAAGTTCATGCTCCGTGCTAAACGTGCAAGTCGT	GAAGGTCGGAGTCAACGGATTCGTGCTAAACGTGCAAGTCGC	CAAATCGCCTCAAGGCATCGTTTGTT
KASP_10.159	6+9K RosBREED SNP chip (RosBREED_snp_sweet_4_07944355)	10158864	T/C	T/T	GGACTGCAACTCCACAAGTTGTGCAAGCAATGTCAATACTGTTTGTCCTCCTAAGTTGTCAGTGAAAGGATCAGATGGGAGTGTGATTGGCTGCAAAAGTG[T/C]ATGCATGGCTTTA**AATCAGCCCCAATATTGTTGCACTG**GTGCTTATGGCTCCCCAGATACATGCCCTCCTACTAACTATTCAAAGATCTTCAAGAACCAGT	T	C	GAAGGTGACCAAGTTCATGCTGAGTGTGATTGGCTGCAAAAGTGT	GAAGGTCGGAGTCAACGGATTGTGTGATTGGCTGCAAAAGTGC	CAGTGCAACAATATTGGGGCTGATT
