# Supplementary material for: Plant N-acylethanolamines play a crucial role in defense and its variation in response to elevated CO2 and temperature in tomato
Source: Hortic Res. 2022 Oct 26;10(1):uhac242. doi: 10.1093/hr/uhac242 (PMC10108025; doi:10.1093/hr/uhac242)
Supplement: Web_Material_uhac242 [file web_material_uhac242.zip › Table. S5.pdf]

Table S5. Comparison of parental genetic maps within the Population #1 (genotyped with the RosBREED cherry 6K SNP array in Castède et al. 2014) and Population #2 (RosBREED cherry 6+9K SNP array).

	SNP array used for genotyping	Parental map	LG1	LG2	LG3	LG4	LG5	LG6	LG7	LG8	**Total***
Number of SNP markers	6K	'Regina'	21	22	15	19	13	16	16	20	142
		'Garnet'	33	18	16	14	10	16	11	19	137
	6+9K	'Regina'	78	82	63	56	92	61	78	88	598
		'Garnet'	183	44	48	33	23	43	17	55	446
Genetic length (cM)	6K	'Regina'	124.2	76.7	69.3	59.9	67.7	81.7	57.6	67.0	604.1
		'Garnet'	151.2	104.7	100.3	95.2	67.4	114	55.9	72.1	760.8
	6+9K	'Regina'	136.0	67.3	74.0	64.4	53.5	90.2	61.5	67.6	614.5
		'Garnet'	167.4	104.2	67.1	76.9	38.3	41.0	51.5	72.6	619.0
Average distance between markers (cM)	6K	'Regina'	6.2	3.7	4.9	3.3	5.6	5.5	3.8	3.5	4.6
		'Garnet'	4.7	6.2	6.7	7.3	7.5	7.6	5.6	4.0	6.2
	6+9K	'Regina'	1.7	0.8	1.2	1.1	0.6	1.5	0.8	0.8	1.1
		'Garnet'	0.9	2.4	1.4	2.3	1.7	0.9	3.0	1.3	1.7
Largest gap size (cM)	6K	'Regina'	25.5	12.8	16.3	11.8	13.5	24.1	12.8	11.7	25.5
		'Garnet'	24.1	27.0	25.6	38.73	30.1	51.5	15.1	10.6	51.5
	6+9K	'Regina'	19.7	6.6	7.6	10.8	6.1	35.5	3.4	8.4	35.5
		'Garnet'	11.0	32.3	13.6	31.1	7.1	7.3	11.3	8.5	32.3

Number of SNPs per linkage group (LG), LGs genetic length, average distance between markers and size of largest gap are presented. *Total number of markers; total genetic length; average distance across all the LGs; largest gap across all the LGs. In grey, genetic maps from Castède et al. 2014.
