# Supplementary material for: Plant N-acylethanolamines play a crucial role in defense and its variation in response to elevated CO2 and temperature in tomato
Source: Hortic Res. 2022 Oct 26;10(1):uhac242. doi: 10.1093/hr/uhac242 (PMC10108025; doi:10.1093/hr/uhac242)
Supplement: Web_Material_uhac242 [file web_material_uhac242.zip › Table. S6.pdf]

**Table S6.** QTLs for flowering date (FD) with Population #1 (single-year and multi-year analyses and all PVE values included).

| Year | QTL name | LG | L (cM) | CI 95% (cM) | Physical position (Mb) | LOD | PVE (%) | d |
|---|---|---|---|---|---|---|---|---|
| 2008* | *qP-FD3.1* | R3 | 33.4 | 6.2-60.6 | 2.40-25.67 | 2.7 | 5.5 | -1.9 |
| | *qP-FD4.1* $^{m}$ | R4 | 22.0 | 16.6-27.3 | 6.67-11.12 | 12.1 | 30.5 | 4.6 |
| | *qP-FD6.1* $^{m}$ | R6 | 75.7 | 52.9-81.7 | 15.28-31.64 | 4.2 | 9.1 | -2.5 |
| | *qP-FD7.1* $^{m}$ | R7 | 6.3 | 0.0-22.5 | 0.48-20.92 | 3.4 | 6.9 | -2.0 |
| | *qP-FD1.2* $^{m}$ | G1 | 109.6 | 62.4-151.2 | 24.09-54.07 | 4.3 | 17.1 | 3.1 |
| 2009* | *qP-FD1.1* $^{m}$ | R1 | 19.0 | 0.0-52.2 | 0.48-27.90 | 3.7 | 5.9 | -1.5 |
| | *qP-FD4.1* $^{m}$ | R4 | 22.8 | 18.5-27.2 | 8.99-11.12 | 24.1 | 45.8 | 4.4 |
| | *qP-FD6.1* $^{m}$ | R6 | 71.7 | 61.8-81.6 | 15.28-31.64 | 7.7 | 10.5 | -2.1 |
| | *qP-FD7.1* $^{m}$ | R7 | 55.9 | 45.3-57.6 | 24.05-28.17 | 6.6 | 8.5 | 1.8 |
| 2010* | *qP-FD4.1* $^{m}$ | R4 | 21.5 | 15.7-27.2 | 6.67-15.68 | 15.3 | 42.1 | 2.0 |
| | *qP-FD7.1* $^{m}$ | R7 | 34.9 | 0.0-57.6 | 0.48-28.17 | 2.7 | 5.9 | 0.1 |
| 2011* | *qP-FD4.1* $^{m}$ | R4 | 20.0 | 13.1-26.9 | 6.67-11.12 | 13.7 | 36.7 | 2.8 |
| | *qP-FD6.1* $^{m}$ | R6 | 66.6 | 23.4-81.7 | 7.07-31.64 | 3.1 | 6.8 | -1.2 |
| | *qP-FD6.2* $^{m}$ | G6 | 24.3 | 6.6-42.1 | 3.07-9.53 | 4.8 | 14.4 | -1.8 |
| 2012* | *qP-FD4.1* $^{m}$ | R4 | 21.6 | 18.2-25.1 | 8.99-11.12 | 12.5 | 36.2 | 2.3 |
| 2013 | *qP-FD4.1* $^{m}$ | R4 | 22.3 | 18.2-26.3 | 8.99-11.12 | 11.4 | 29.4 | 1.7 |
| | *qP-FD1.2* $^{m}$ | G1 | 102.1 | 6.9-151.2 | 1.91-54.07 | 3.6 | 14.2 | 0.6 |
| 2014 | *qP-FD4.1* $^{m}$ | R4 | 19.7 | 12.7-26.6 | 6.67-11.12 | 14.9 | 38.0 | 3.3 |
| | *qP-FD7.1* $^{m}$ | R7 | 41.5 | 0.0-57.6 | 0.48-28.17 | 3.4 | 7.0 | 0.7 |
| 2015 | *qP-FD4.1* $^{m}$ | R4 | 20.2 | 14.1-26.4 | 6.67-11.12 | 18.4 | 46.3 | 2.3 |
| | *qP-FD7.1* $^{m}$ | R7 | 27.5 | 0.0-57.6 | 0.48-28.17 | 3.3 | 6.1 | -0.1 |
| 2016 | *qP-FD4.1* $^{m}$ | R4 | 19.7 | 12.3-27.0 | 6.67-11.12 | 10.8 | 30.1 | 3.9 |
| | *qP-FD7.1* $^{m}$ | R7 | 24.5 | 0.0-57.6 | 0.48-28.17 | 4.3 | 10.6 | -0.7 |
| | *qP-FD1.2* $^{m}$ | G1 | 99.6 | 1.0-151.2 | 0.35-54.07 | 3.4 | 10.3 | 0.6 |
| | *qP-FD6.2* $^{m}$ | G6 | 19.5 | 3.3-35.7 | 1.06-8.52 | 4.1 | 14.6 | -2.6 |
| 2017 | *qP-FD4.1* $^{m}$ | R4 | 19.7 | 14.1-25.3 | 6.67-11.12 | 11.7 | 32.4 | 2.9 |
| | *qP-FD7.1* $^{m}$ | R7 | 34.5 | 0.0-57.6 | 0.48-28.17 | 3.0 | 6.9 | 0.2 |
| | *qP-FD1.2* $^{m}$ | G1 | 93.0 | 0.0-151.2 | 0.35-54.07 | 3.3 | 13 | 0.3 |
| **Multi-Year** | **QTL name** | **LG** | **L (cM)** | **CI 95% (cM)** | **Physical position (Mb)** | **LOD** | **PVE mean (%)** | **d mean** |
| | *qP-FD1.1* $^{m}$ | R1 | 12.8 | 0.0-32.7 | 0.48-11.89 | 18.9 | 4.0 | -0.9 |
| | *qP-FD2.1* $^{m}$ | R2 | 29.8 | 27.2-32.3 | 26.33-27.77 | 19.5 | 3.7 | -0.9 |
| | *qP-FD4.1* $^{m}$ | R4 | 20.6 | 19-22.2 | 8.99-11.12 | 146.1 | 34.3 | 2.9 |
| | *qP-FD5.1* $^{m}$ | R5 | 19.0 | 0.0-42.4 | 3.30-14.88 | 11.4 | 2.1 | 0.6 |
| | *qP-FD6.1* $^{m}$ | R6 | 66.4 | 36.4-81.7 | 10.96-31.64 | 18.7 | 3.4 | -0.8 |
| | *qP-FD7.1* $^{m}$ | R7 | 54.6 | 34.7-57.6 | 22.16-28.17 | 31.9 | 5.7 | 1.1 |
| | *qP-FD8.1* $^{m}$ | R8 | 18.1 | 0.0-43.3 | 1.91-18.94 | 8.8 | 1.6 | -0.4 |
| | *qP-FD1.2* $^{m}$ | G1 | 128.7 | 101.7-151.2 | 39.91-54.07 | 24.1 | 7.3 | 1.3 |
| | *qP-FD2.2* $^{m}$ | G2 | 15.3 | 3.8-26.7 | 2.59-13.18 | 17.2 | 4.7 | -1.0 |
| | *qP-FD3.1* $^{m}$ | G3 | 88.8 | 32.0-100.3 | 6.96-29.84 | 12.2 | 3.4 | 0.7 |
| | *qP-FD5.2* $^{m}$ | G5 | 11.3 | 0.0-25.6 | 6.82-9.10 | 11.4 | 3.1 | -0.8 |
| | *qP-FD6.2* $^{m}$ | G6 | 20.7 | 16.9-24.4 | 3.07-5.79 | 38.5 | 12.8 | -1.9 |
| | *qP-FD8.2* $^{m}$ | G8 | 58.0 | 29.1-72.1 | 12.40-22.82 | 12.2 | 3.1 | 0.9 |

*, data from Castède et al. 2014; LG, linkage group; L, distance from the beginning of the chromosome to the point of maximum LOD in the interval; CI, confidence interval; Physical position of flanking markers on 'Regina' v1 genome sequence; LOD, logarithm of the odds ratio; PVE, phenotypic variance explained by the QTL in percentage of the total variation; PVE mean, mean value of PVE in the multi-environment analysis; d, difference X(A) – X(B) according to the year of evaluation, where A and B are the two homozygotes at the marker loci; (+/ ), the sign varies according to the year of evaluation; d mean, mean value of d in the multi-environment analysis. QTLs detected every year are shaded in grey.
