# Supplementary material for: Plant N-acylethanolamines play a crucial role in defense and its variation in response to elevated CO2 and temperature in tomato
Source: Hortic Res. 2022 Oct 26;10(1):uhac242. doi: 10.1093/hr/uhac242 (PMC10108025; doi:10.1093/hr/uhac242)
Supplement: Web_Material_uhac242 [file web_material_uhac242.zip › Table. S7.pdf]

Table S7. QTLs for flowering date (FD) with Population #2 (single-year and multi-year analyses and all PVE values included)

Year	QTL name	LG	L (cM)	CI 95% (cM)	Physical position (Mb)	LOD	PVE (%)	d
2018	*qP-FD1.1* ^{m}	R1	23.0	6.5-39.5	0.68-13.49	5.8	2.8	0.9
	qP-FD2.1 ^{m}	R2	24.2	9.9-38.5	20.51-30.93	4.7	2.3	0.8
	qP-FD3.2	R3	68.9	41.1-73.3	18.57-30.11	3.2	1.5	-0.6
	qP-FD4.1 ^{m}	R4	26.7	25.4-28.0	9.78-10.16	55.0	35.0	-3.2
	qP-FD5.1 ^{m}	R5	31.0	9.6-52.4	1.15-19.15	8.4	4.2	1.1
	qP-FD6.1 ^{m}	R6	63.6	17.4-89.8	6.64-32.84	5.9	3.5	-1.0
	qP-FD7.1 ^{m}	R7	50.5	29.2-61.6	20.81-29.42	7.1	3.5	-0.9
	qP-FD1.2 ^{m}	G1	154.0	139.2-167.7	45.66-54.15	9.7	8.8	-1.7
	qP-FD2.2 ^{m}	G2	28.4	0.0-62.7	0.53-30.96	3.7	3.1	-0.9
	qP-FD7.2 ^{m}	G7	9.0	0.0-27.99	0.36-18.75	3.0	2.5	0.9
	qP-FD8.2 ^{m}	G8	25.3	3.4-47.2	1.65-17.68	3.5	3.0	0.9
2019	*qP-FD1.1* ^{m}	R1	28.7	17.8-55.4	9.85-27.90	7.5	3.4	1.2
	qP-FD2.1 ^{m}	R2	33.7	12.1-55.4	22.92-35.14	4.5	2.0	0.9
	qP-FD4.1 ^{m}	R4	27.2	25.5-28.8	9.78-10.33	64.8	39.8	-4.1
	qP-FD5.1 ^{m}	R5	24.3	1.1-47.6	3.32-17.64	7.1	3.2	1.2
	qP-FD6.1 ^{m}	R6	39.8	6.5-73.0	3.81-27.02	8.7	4.4	-1.4
	qP-FD7.1 ^{m}	R7	52.4	19.6-61.6	18.41-29.42	6.0	2.7	-0.9
	qP-FD8.3 ^{m}	R8	63.3	51.6-69.0	19.38-26.47	5.3	2.4	-1.0
	qP-FD1.2 ^{m}	G1	151.0	100.5-167.7	36.13-54.15	4.6	4.5	-1.4
2021	*qP-FD1.1* ^{m}	R1	28.7	15.6-41.9	9.85-13.49	9.2	4.5	1.2
	qP-FD2.1 ^{m}	R2	21.6	8.7-34.4	20.51-29.81	4.3	2.0	0.8
	qP-FD4.1 ^{m}	R4	27.3	25.3-29.4	9.78-10.87	60.2	37.8	-3.6
	qP-FD5.1 ^{m}	R5	43.4	31.7-53.6	14.10-19.20	5.0	2.4	0.9
	qP-FD6.1 ^{m}	R6	69.7	43.4-89.8	18.26-32.84	4.1	2.2	-0.8
	qP-FD7.1 ^{m}	R7	44.9	1.3-61.6	23.56-29.42	7.2	3.4	-0.7
	qP-FD8.3 ^{m}	R8	54.6	43.0-66.2	17.49-25.20	7.7	3.7	-1.1
	qP-FD1.2 ^{m}	G1	152.5	118.2-167.7	41.72-54.15	5.5	5.1	-1.3
	qP-FD2.2 ^{m}	G2	42.0	0.0-103.0	0.53-38.27	5.2	4.7	-0.6
	qP-FD7.2 ^{m}	G7	9.2	0.00-25.3	0.36-18.75	3.5	3.1	1.1
**Multi-Year**	**QTL name**	**LG**	**L (cM)**	**CI 95% (cM)**	**Physical position (Mb)**	**LOD**	**PVE mean (%)**	**d mean**
	qP-FD1.1 ^{m}	R1	26.9	21.2-32.6	9.85-11.96	21.1	3.3	1.1
	qP-FD2.1 ^{m}	R2	23.1	15.8-30.5	23.72-27.82	12.3	1.9	0.8
	qP-FD4.1 ^{m}	R4	26.9	< 0.5 cM	9.78-10.16	180.3	37.1	-3.6
	qP-FD5.1 ^{m}	R5	35.9	20.1-51.7	10.27-18.77	17.8	2.8	1.0
	qP-FD6.1 ^{m}	R6	55.1	25.2-84.9	9.59-30.27	16.2	3.5	-1.1
	qP-FD7.1 ^{m}	R7	53.8	36.2-61.6	23.74-29.42	19.7	3.0	-1.0
	qP-FD8.3 ^{m}	R8	60.7	53.9-67.4	20.01-25.42	14.1	2.2	-0.8
	qP-FD1.2 ^{m}	G1	155.5	150.1-160.9	49.13-50.88	20.0	6.1	-1.5
	qP-FD2.2 ^{m}	G2	33.4	0.0-84.2	0.53-33.79	9.8	2.9	-0.7
	qP-FD7.2 ^{m}	G7	8.0	0.0-17.2	0.36-12.88	7.6	2.2	0.8
	qP-FD8.2 ^{m}	G8	26.7	8.3-45.1	2.45-17.36	6.0	1.7	0.7

LG, linkage group; L, distance from the beginning of the chromosome to the point of maximum LOD in the interval; CI, confidence interval; Physical position of flanking markers on 'Regina' v1 genome sequence; LOD, logarithm of the odds ratio; PVE, phenotypic variance explained by the QTL in percentage of the total variation; PVE mean, mean value of PVE in the multi-environment analysis; d, difference X(A) – X(B) according to the year of evaluation, where A and B are the two homozygotes at the marker loci; (+/), the sign varies according to the year of evaluation; d mean, mean value of d in the multi-environment analysis. QTLs detected every year are shaded in grey.
