# Supplementary material for: Plant N-acylethanolamines play a crucial role in defense and its variation in response to elevated CO2 and temperature in tomato
Source: Hortic Res. 2022 Oct 26;10(1):uhac242. doi: 10.1093/hr/uhac242 (PMC10108025; doi:10.1093/hr/uhac242)
Supplement: Web_Material_uhac242 [file web_material_uhac242.zip › Table. S8.pdf]

Table S8. Genotypes at the 17 KASPs and Full flowering (FF) values (in Julian Days) in 2018, 2019 and 2021 and lsmeans of the individuals of the Population #2

KASP	KASP_9.269	KASP_9.271	KASP_9.456	KASP_9.727	KASP_9.780	KASP_9.781	KASP_9.801	KASP_9.814	KASP_9.890	KASP_9.916	KASP_9.933	KASP_9.935	KASP_9.936	KASP_9.958	KASP_9.970	KASP_10.090	KASP_10.159	N	Flowering date			
Position (bp)	9 268 774	9 270 618	9 455 997	9 726 870	9 780 346	9 780 552	9 800 899	9 813 748	9 889 761	9 915 521	9 932 706	9 935 374	9 935 681	9 957 746	9 970 195	10 089 579	10 158 864		FF 2018	FF 2019	FF 2021	FFlsmeans
GARNET	T/T	A/A	A/A	A/A	A/A	C/C	A/A	G/G	C/C	G/G	C/C	G/G	A/A	C/C	A/A	A/A	T/T	G	99.0	88.0	85.0	90.7
REGINA	C/T	G/A	A/T	A/G	A/G	C/G	A/G	G/A	A/C	G/A	C/T	A/G	A/G	C/T	A/T	A/G	T/C	R	106.0	101.0	95.0	100.7
Non-Rec like Garnet	T/T	A/A	A/A	A/A	A/A	C/C	A/A	G/G	C/C	G/G	C/C	G/G	A/A	C/C	A/A	A/A	T/T	641	97.4	88.4	85.8	90.5
Non-Rec like Regina	C/T	G/A	A/T	A/G	A/G	C/G	A/G	G/A	A/C	G/A	C/T	A/G	A/G	C/T	A/T	A/G	T/C	697	99.6	92.1	89.0	93.6
Rec #1 (ind. 1)	C/T	G/A	A/A	A/A	A/A	C/C	A/A	G/G	C/C	G/G	C/C	G/G	A/A	C/C	A/A	A/A	T/T	1	102.0	93.0	90.0	95.0
Rec #1 (ind. 2)	C/T	G/A	A/A	A/A	A/A	C/C	A/A	G/G	C/C	G/G	C/C	G/G	A/A	C/C	A/A	A/A	T/T	1	96.0	86.0	84.0	88.7
Rec #2 (ind. 3)	T/T	A/A	A/T	A/G	A/G	C/G	A/G	G/A	A/C	G/A	C/T	A/G	A/G	C/T	A/T	A/G	T/C	1	101.0	89.0	86.0	92.0
Rec #3 (ind. 4)	C/T	G/A	A/T	A/A	A/A	C/C	A/A	G/G	C/C	G/G	C/C	G/G	A/A	C/C	A/A	A/A	T/T	1	96.0	90.0	83.0	89.7
Rec #3 (ind. 5)	C/T	G/A	A/T	A/A	A/A	C/C	A/A	G/G	C/C	G/G	C/C	G/G	A/A	C/C	A/A	A/A	T/T	1	97.0	89.0	86.0	93.3
Rec #3 (ind. 6)	C/T	G/A	A/T	A/A	A/A	C/C	A/A	G/G	C/C	G/G	C/C	G/G	A/A	C/C	A/A	A/A	T/T	1	97.0	86.0	85.0	90.7
Rec #3 (ind. 7)	C/T	G/A	A/T	A/A	A/A	C/C	A/A	G/G	C/C	G/G	C/C	G/G	A/A	C/C	A/A	A/A	T/T	1	102.0	91.0	91.0	89.3
Rec #3 (ind. 8)	C/T	G/A	A/T	A/A	A/A	C/C	A/A	G/G	C/C	G/G	C/C	G/G	A/A	C/C	A/A	A/A	T/T	1	98.0	90.0	87.0	94.7
Rec #3 (ind. 9)	C/T	G/A	A/T	A/A	A/A	C/C	A/A	G/G	C/C	G/G	C/C	G/G	A/A	C/C	A/A	A/A	T/T	1	95.0	86.0	83.0	91.7
Rec #3 (ind. 10)	C/T	G/A	A/T	A/A	A/A	C/C	A/A	G/G	C/C	G/G	C/C	G/G	A/A	C/C	A/A	A/A	T/T	1	96.0	88.0	84.0	88.0
Rec #3 (ind. 11)	C/T	G/A	A/T	A/A	A/A	C/C	A/A	G/G	C/C	G/G	C/C	G/G	A/A	C/C	A/A	A/A	T/T	1	97.0	89.0	89.0	89.3
Rec #3 (ind. 12)	C/T	G/A	A/T	A/A	A/A	C/C	A/A	G/G	C/C	G/G	C/C	G/G	A/A	C/C	A/A	A/A	T/T	1	96.0	89.0	83.0	91.7
Rec #3 (ind. 13)	C/T	G/A	A/T	A/A	A/A	C/C	A/A	G/G	C/C	G/G	C/C	G/G	A/A	C/C	A/A	A/A	T/T	1	103.0	91.0	86.0	89.3
Rec #4 (ind. 14)	T/T	A/A	A/A	A/G	A/G	C/G	A/G	G/A	A/C	G/A	C/T	A/G	A/G	C/T	A/T	A/G	T/C	1	98.0	91.0	88.0	92.3
Rec #4 (ind. 15)	T/T	A/A	A/A	A/G	A/G	C/G	A/G	G/A	A/C	G/A	C/T	A/G	A/G	C/T	A/T	A/G	T/C	1	99.0	93.0	88.0	93.3
Rec #4 (ind. 16)	T/T	A/A	A/A	A/G	A/G	C/G	A/G	G/A	A/C	G/A	C/T	A/G	A/G	C/T	A/T	A/G	T/C	1	97.0	91.0	90.0	92.7
Rec #4 (ind. 17)	T/T	A/A	A/A	A/G	A/G	C/G	A/G	G/A	A/C	G/A	C/T	A/G	A/G	C/T	A/T	A/G	T/C	1	98.0	90.0	88.0	92.0
Rec #4 (ind. 18)	T/T	A/A	A/A	A/G	A/G	C/G	A/G	G/A	A/C	G/A	C/T	A/G	A/G	C/T	A/T	A/G	T/C	1	99.0	92.0	90.0	93.7
Rec #4 (ind. 19)	T/T	A/A	A/A	A/G	A/G	C/G	A/G	G/A	A/C	G/A	C/T	A/G	A/G	C/T	A/T	A/G	T/C	1	101.0	95.0	90.0	95.3
Rec #4 (ind. 20)	T/T	A/A	A/A	A/G	A/G	C/G	A/G	G/A	A/C	G/A	C/T	A/G	A/G	C/T	A/T	A/G	T/C	1	101.0	93.0	92.0	95.3
Rec #5 (ind. 21)	T/T	A/A	A/A	A/A	A/A	C/C	A/G	G/A	A/C	G/A	C/T	A/G	A/G	C/T	A/T	A/G	T/C	1	97.0	90.0	89.0	92.0
Rec #6 (ind. 22)	C/T	G/A	A/T	A/G	A/G	C/G	A/G	G/A	C/C	G/G	C/C	G/G	A/A	C/C	A/A	A/A	T/T	1	95.0	84.0	85.0	88.0
Rec #6 (ind. 23)	C/T	G/A	A/T	A/G	A/G	C/G	A/G	G/A	C/C	G/G	C/C	G/G	A/A	C/C	A/A	A/A	T/T	1	98.0	89.0	87.0	91.3
Rec #6 (ind. 24)	C/T	G/A	A/T	A/G	A/G	C/G	A/G	G/A	C/C	G/G	C/C	G/G	A/A	C/C	A/A	A/A	T/T	1	98.0	90.0	89.0	92.3
Rec #6 (ind. 25)	C/T	G/A	A/T	A/G	A/G	C/G	A/G	G/A	C/C	G/G	C/C	G/G	A/A	C/C	A/A	A/A	T/T	1	98.0	88.0	87.0	91.0
Rec #7 (ind. 26)	T/T	A/A	A/A	A/A	A/A	C/C	A/A	G/G	A/C	G/A	C/T	A/G	A/G	C/T	A/T	A/G	T/C	1	NA	91.0	87.0	92.2
Rec #7 (ind. 27)	T/T	A/A	A/A	A/A	A/A	C/C	A/A	G/G	A/C	G/A	C/T	A/G	A/G	C/T	A/T	A/G	T/C	1	100.0	90.0	88.0	92.7
Rec #7 (ind. 28)	T/T	A/A	A/A	A/A	A/A	C/C	A/A	G/G	A/C	G/A	C/T	A/G	A/G	C/T	A/T	A/G	T/C	1	99.0	88.0	85.0	90.7
Rec #7 (ind. 29)	T/T	A/A	A/A	A/A	A/A	C/C	A/A	G/G	A/C	G/A	C/T	A/G	A/G	C/T	A/T	A/G	T/C	1	97.0	89.0	85.0	90.3
Rec #8 (ind. 30)	C/T	G/A	A/T	A/G	A/G	C/G	A/G	G/A	A/C	G/G	C/C	G/G	A/A	C/C	A/A	A/A	T/T	1	99.0	89.0	85.0	91.0
Rec #8 (ind. 31)	C/T	G/A	A/T	A/G	A/G	C/G	A/G	G/A	A/C	G/G	C/C	G/G	A/A	C/C	A/A	A/A	T/T	1	100.0	90.0	86.0	92.0
Rec #8 (ind. 32)	C/T	G/A	A/T	A/G	A/G	C/G	A/G	G/A	A/C	G/G	C/C	G/G	A/A	C/C	A/A	A/A	T/T	1	97.0	89.0	87.0	91.0
Rec #9 (ind. 33)	T/T	A/A	A/A	A/A	A/A	C/C	A/A	G/G	C/C	G/A	C/T	A/G	A/G	C/T	A/T	A/G	T/C	1	98.0	91.0	86.0	91.7
Rec #9 (ind. 34)	T/T	A/A	A/A	A/A	A/A	C/C	A/A	G/G	C/C	G/A	C/T	A/G	A/G	C/T	A/T	A/G	T/C	1	100.0	95.0	88.0	94.3
Rec #9 (ind. 35)	T/T	A/A	A/A	A/A	A/A	C/C	A/A	G/G	C/C	G/A	C/T	A/G	A/G	C/T	A/T	A/G	T/C	1	103.0	95.0	90.0	96.0
Rec #9 (ind. 36)	T/T	A/A	A/A	A/A	A/A	C/C	A/A	G/G	C/C	G/A	C/T	A/G	A/G	C/T	A/T	A/G	T/C	1	102.0	90.0	93.0	95.0
Rec #9 (ind. 37)	T/T	A/A	A/A	A/A	A/A	C/C	A/A	G/G	C/C	G/A	C/T	A/G	A/G	C/T	A/T	A/G	T/C	1	103.0	92.0	90.0	95.0
Rec #9 (ind. 38)	T/T	A/A	A/A	A/A	A/A	C/C	A/A	G/G	C/C	G/A	C/T	A/G	A/G	C/T	A/T	A/G	T/C	1	100.0	90.0	87.0	92.3
Rec #10 (ind. 39)	C/T	G/A	A/T	A/G	A/G	C/G	A/G	G/A	A/C	G/A	C/T	G/G	A/A	C/C	A/A	A/A	T/T	1	97.0	90.0	86.0	91.0
Rec #11 (ind. 40)	C/T	G/A	A/T	A/G	A/G	C/G	A/G	G/A	A/C	G/A	C/T	A/G	A/G	C/C	A/A	A/A	T/T	1	99.0	90.0	89.0	92.7
Rec #12 (ind. 41)	C/T	G/A	A/T	A/G	A/G	C/G	A/G	G/A	A/C	G/A	C/T	A/G	A/G	C/T	A/T	A/A	T/T	1	103.0	91.0	90.0	94.7

FF values in 2018, 2019 and 2021 and lsmeans are presented for the parental cultivars 'Garnet' (G) and 'Regina' (R), the non-recombinant individuals (Non-Rec #like G and Non-Rec #like R) and the recombinant s (Rec #1 to Rec #12). 'Garnet' is homozygous for all markers, 'Regina' is heterozygous for all markers. The number of individuals is presented in column 'N'. The physical position of the seventeen KASP markers on the 'Regina' genome is given in line 'Position (bp)'. In FD columns, a scale color from red (early FD) to blue (late FD) was used for a better visualization.
