# Supplementary material for: Plant N-acylethanolamines play a crucial role in defense and its variation in response to elevated CO2 and temperature in tomato
Source: Hortic Res. 2022 Oct 26;10(1):uhac242. doi: 10.1093/hr/uhac242 (PMC10108025; doi:10.1093/hr/uhac242)
Supplement: Web_Material_uhac242 [file web_material_uhac242.zip › Table. S9.pdf]

Table S9. List of the 65 predicted candidates genes located within the 380 kb-interval of the QTL on LG4 of 'Regina'.

Sweet cherry gene ID	Expressed in 'Regina' and 'Garnet' ? (1)	Start (bp) (2)	End (bp) (2)	*Prunus persica* match (Peach match)	*Arabidopsis thaliana* match	Description (Blast2GO)	If unknown/ hypothetical protein, TAIR description	E-value	Gene Ontology (Number of Hits)	Gene ontology Names (C: cellular components, F: molecular functions, P: biological processes)
PAV04_REGINAg0203151	YES	9 778 898	9 780 665	Prupe.4G138100.1	AT2G33350	CONSTANS-LIKE 3 (COL3)		0.0E0	2	C:nucleus; F:protein binding
PAV04_REGINAg0203161	NO	9 785 276	9 785 873	Prupe.2G165000.1	AT5G08130	Unknown protein	Basic helix-loop-helix (bHLH) DNA-binding superfamily protein (BIM1)			
PAV04_REGINAg0203171	YES	9 786 702	9 790 244	Prupe.4G138200.1	AT1G31320	LOB domain-containing 4-like (LBD4)		6.8E-108		
PAV04_REGINAg0203181	YES	9 800 093	9 801 841	Prupe.4G138300.1	AT3G12900	Feruloyl CoA ortho-hydroxylase 1 (2-ODD superfamily protein)		0.0E0	3	F:dioxygenase activity; F:metal ion binding; P:oxidation-reduction process
PAV04_REGINAg0203191	YES	9 803 531	9 816 297	Prupe.4G138400.1	AT1G80190	Molybdenum cofactor sulfurase ABA3		0.0E0	7	F:molybdenum cofactor sulfurtransferase activity; F:pyridoxal phosphate binding; F:Mo-molybdopterin cofactor sulfurase activity; F:molybdenum ion binding; F:lyase activity; P:Mo-molybdopterin cofactor biosynthetic process; C:molybdopterin synthase complex
PAV04_REGINAg0203201	YES	9 823 361	9 825 594	Prupe.4G138500.1	AT3G04410	JUNGBRUNNEN 1-like transcription factor (JUB1)		0.0E0	5	F:GTP binding; C:nucleus; F:DNA binding; F:GTPase activity; P:regulation of transcription, DNA-templated
PAV04_REGINAg0203211	YES	9 834 512	9 839 671	Prupe.4G138800.1	AT1G16560	Post-GPI attachment to proteins factor 3 (PGAP3)		0.0E0	5	F:hydrolase activity, acting on ester bonds; P:GPI anchor biosynthetic process; C:integral component of membrane; C:Golgi membrane; C:intrinsic component of endoplasmic reticulum membrane
PAV04_REGINAg0203221	YES	9 840 369	9 844 938	Prupe.4G138700.1	AT1G56070	Elongation factor 2 (eEF2)		0.0E0	11	F:GTP binding; C:nucleus; F:DNA binding; P:regulation of translational elongation; F:translation elongation factor activity; F:GTPase activity; F:ribosome binding; C:cytosol; P:regulation of transcription, DNA-templated; C:ribonucleoprotein complex; C:ribosome
PAV04_REGINAg0203231	YES	9 845 942	9 849 788	Prupe.4G138900.1	AT1G56070	Elongation factor 2 (eEF2)		0.0E0	11	F:GTP binding; C:nucleus; F:DNA binding; P:regulation of translational elongation; F:translation elongation factor activity; F:GTPase activity; F:ribosome binding; C:cytosol; P:regulation of transcription, DNA-templated; C:ribonucleoprotein complex; C:ribosome
PAV04_REGINAg0203241	YES	9 851 946	9 853 807	Prupe.4G141800.1	AT1G16590	DNA polymerase zeta processivity subunit-like isoform X1		7.1E-174	4	F:DNA binding; C:zeta DNA polymerase complex; P:cellular response to DNA damage stimulus; P:response to UV-B
PAV04_REGINAg0203251	NO	9 865 781	9 866 293	Prupe.5G057200.1	AT4G28940	Proline iminopeptidase-like		5.9E-59		
PAV04_REGINAg0203281	YES	9 874 912	9 876 861	Prupe.4G139300.1	AT1G79120	ROOT PRIMORDIUM DEFECTIVE 1 (RPD1)		0.0E0		

PAV04_REGINAg0203291	YES	9 888 563	9 890 443	Prupe.4G139400.1	AT2G12290	Probable BOI-related E3 ubiquitin-protein ligase 3	0.0E0	4	C:nucleus; F:ubiquitin-protein transferase activity; P:regulation of programmed cell death; P:protein ubiquitination
PAV04_REGINAg0203301	YES	9 899 712	9 901 063	Prupe.4G139500.1	AT4G27290	G-type lectin S-receptor-like serine threonine-kinase (GsSRK)	1.3E-115	7	F:ATP binding; P:recognition of pollen; C:integral component of membrane; P:serine family amino acid metabolic process; F:protein serine/threonine kinase activity; P:protein phosphorylation; C:plasma membrane
PAV04_REGINAg0203321	YES	9 901 340	9 903 275	Prupe.4G139500.1	AT4G27290	G-type lectin S-receptor-like serine threonine-kinase (GsSRK)	0.0E0	7	F:ATP binding; P:recognition of pollen; C:integral component of membrane; P:serine family amino acid metabolic process; F:protein serine/threonine kinase activity; P:protein phosphorylation; C:plasma membrane
PAV04_REGINAg0203341	YES	9 906 269	9 910 099	Prupe.4G139500.1	AT4G27290	G-type lectin S-receptor-like serine threonine-kinase (GsSRK)	0.0E0	7	F:ATP binding; P:recognition of pollen; C:integral component of membrane; P:serine family amino acid metabolic process; F:protein serine/threonine kinase activity; P:protein phosphorylation; C:plasma membrane
PAV04_REGINAg0203351	YES	9 910 896	9 915 249	Prupe.4G139600.1	AT3G12940	Isopenicillin N synthase (IPNS)	0.0E0	5	F:ATP binding; C:integral component of membrane; F:protein kinase activity; P:protein phosphorylation; F:hydrolase activity
PAV04_REGINAg0203361	YES	9 915 252	9 918 357	Prupe.4G139600.1	AT3G12930	Protein Iojap/ribosomal silencing factor (RsfS)	3.3E-178	4	C:chloroplast; F:ribosomal large subunit binding; P:negative regulation of translation; P:negative regulation of ribosome biogenesis
PAV04_REGINAg0203371	YES	9 919 147	9 924 461	Prupe.4G139800.1	AT1G16610	Serine/arginine-rich splicing factor SR45a	0.0E0	5	C:ASAP complex; F:RNA binding; C:cytoplasm; P:regulation of alternative mRNA splicing, via spliceosome; C:nucleoplasm
PAV04_REGINAg0203381	YES	9 924 669	9 930 010	Prupe.4G139900.1	AT3G12950	Peptidase family S1 (PA clan)	0.0E0	1	F:RNA binding
PAV04_REGINAg0203391	YES	9 931 207	9 937 225	Prupe.4G140100.1	AT5G62860	F-box/Kelch-repeat protein	0.0E0	2	F:protein binding; C:integral component of membrane
PAV04_REGINAg0203401	YES	9 937 570	9 938 625	Prupe.4G140200.1	AT4G34770	Auxin-responsive protein SAUR71	8.4E-96	1	P:response to auxin
PAV04_REGINAg0203411	YES	9 940 273	9 946 653	Prupe.4G140300.1	AT1G56050	Ribosome-binding ATPase	0.0E0	7	F:GTP binding; F:ATPase activity; F:ATP binding; C:cytoplasm; F:ribosome binding; F:ribosomal large subunit binding; P:response to auxin
PAV04_REGINAg0203421	YES	9 951 223	9 960 375	Prupe.4G140400.1	AT1G79090	Protein PAT1 homolog (PAT1)	0.0E0	5	F:RNA binding; P:deadenylation-dependent decapping of nuclear-transcribed mRNA; P:cytoplasmic mRNA processing body assembly; C:P-body; F:isomerase activity
PAV04_REGINAg0203431	YES	9 963 583	9 965 615	Prupe.4G140500.1	AT4G38310	Glycosyltransferase (Xyloglucan 6-xylosyltransferase)	0.0E0	5	C:trans-Golgi network; C:endosome; C:integral component of membrane; F:galactosyltransferase activity; F:xyloglucan 6-xylosyltransferase activity
PAV04_REGINAg0203441	YES	9 967 478	9 968 354	Prupe.4G140500.1	AT4G37690	Glycosyltransferase (Xyloglucan 6-xylosyltransferase)	7.0E-69	5	C:trans-Golgi network; C:endosome; C:integral component of membrane; F:galactosyltransferase activity; F:xyloglucan 6-xylosyltransferase activity
PAV04_REGINAg0203451	YES	9 969 773	9 979 872	Prupe.4G140600.1	AT1G04390	BTB POZ domain-containing At1g04390 isoform X1	0.0E0	1	F:protein binding

PAV04_REGINAg0203461	NO	9 985 950	9 986 269	Prupe.4G140700.1	AT5G67160	BAHD acyltransferase At5g47980-like		1.6E-54	1	F:transferase activity, transferring acyl groups other than amino-acyl groups
PAV04_REGINAg0203471	NO	9 986 284	9 987 444	Prupe.4G140700.1	AT2G33860	BAHD acyltransferase At5g47980-like		0.0E0	1	F:transferase activity, transferring acyl groups other than amino-acyl groups
PAV04_REGINAg0203481	YES	9 990 407	9 991 831	Prupe.4G140700.1	AT3G26040	BAHD acyltransferase At5g47980-like		0.0E0	1	F:transferase activity, transferring acyl groups other than amino-acyl groups
PAV04_REGINAg0203491	YES	9 993 503	9 995 317	Prupe.4G140800.1	AT2G28970	BAHD acyltransferase At5g47980-like		8.0E-11(	1	F:transferase activity, transferring acyl groups other than amino-acyl groups
PAV04_REGINAg0203511	YES	10 014 259	10 015 864	Prupe.1G235900.1	AT5G41992	BAHD acyltransferase At5g47980-like		0.0E0	1	F:transferase activity, transferring acyl groups other than amino-acyl groups
PAV04_REGINAg0203521	NO	10 020 759	10 021 049	Prupe.4G140900.1	AT3G12960	Hypothetical protein	-	1.2E-60		
PAV04_REGINAg0203531	YES	10 022 515	10 023 882	Prupe.7G129900.1	AT5G39050	BAHD acyltransferase At5g47980-like		0.0E0	1	F:transferase activity, transferring acyl groups other than amino-acyl groups
PAV04_REGINAg0203541	NO	10 028 103	10 028 393	Prupe.4G140900.1	AT3G12960	Hypothetical protein	-	1.2E-60		
PAV04_REGINAg0203551	YES	10 028 796	10 034 923	Prupe.4G141000.1	AT5G26360	T-complex protein 1 (TCP-1) subunit gamma		0.0E0	4	C:chaperonin-containing T-complex; F:ATP binding; F:unfolded protein binding; P:protein folding
PAV04_REGINAg0203561	NO	10 039 574	10 039 894	Prupe.4G141100.1	AT1G20030	Thaumatin-like protein 1 (TL1)		1.5E-61	2	C:extracellular region; P:defense response
PAV04_REGINAg0203571	NO	10 041 048	10 041 401	Prupe.3G254300.1	AT3G29680	Vinorine synthase-like		5.7E-65	2	F:vinorine synthase activity; P:indole biosynthetic process
PAV04_REGINAg0203581	NO	10 041 492	10 042 169	Prupe.1G354400.1	AT2G10931	Vinorine synthase-like		6.0E-16:	1	F:transferase activity, transferring acyl groups other than amino-acyl groups
PAV04_REGINAg0203601	NO	10 055 670	10 056 824	Prupe.4G142000.1	AT1G21540	Vinorine synthase-like		0.0E0	1	F:transferase activity, transferring acyl groups other than amino-acyl groups
PAV04_REGINAg0203611	YES	10 058 961	10 059 266	Prupe.4G142000.1	AT2G27660	Vinorine synthase-like		4.7E-60	1	F:transferase activity, transferring acyl groups other than amino-acyl groups
PAV04_REGINAg0203621	YES	10 059 895	10 060 968	Prupe.4G140800.1	AT5G09800	Vinorine synthase-like		0.0E0	1	F:transferase activity, transferring acyl groups other than amino-acyl groups
PAV04_REGINAg0203631	YES	10 070 471	10 070 953	Prupe.2G010800.1	AT5G38130	BAHD acyltransferase At5g47980-like		2.6E-11!	1	F:transferase activity, transferring acyl groups other than amino-acyl groups
PAV04_REGINAg0203641	NO	10 071 921	10 072 771	Prupe.1G235900.1	AT4G29250	BAHD acyltransferase At5g47980-like		0.0E0	1	F:transferase activity, transferring acyl groups other than amino-acyl groups
PAV04_REGINAg0203651	YES	10 074 175	10 077 514	Prupe.4G141500.1	AT2G19170	BAHD acyltransferase At5g47980-like		0.0E0	1	F:transferase activity, transferring acyl groups other than amino-acyl groups
PAV04_REGINAg0203661	YES	10 077 674	10 077 952	Prupe.8G099100.1	AT4G30210	Hypothetical protein	P450 reductase 2 ATR2	4.3E-30		
PAV04_REGINAg0203671	NO	10 078 345	10 078 827	Prupe.4G141500.1	AT5G67160	BAHD acyltransferase At5g47980-like		1.3E-94	1	F:transferase activity, transferring acyl groups other than amino-acyl groups
PAV04_REGINAg0203681	NO	10 081 199	10 082 548	Prupe.1G235900.1	AT5G04770	BAHD acyltransferase At5g47980-like		0.0E0	1	F:transferase activity, transferring acyl groups other than amino-acyl groups
PAV04_REGINAg0203701	YES	10 083 238	10 092 221	Prupe.3G114700.1	AT4G18640	Hypothetical protein	Leucine-rich repeat protein kinase family protein MRH1	3.3E-22		
PAV04_REGINAg0203711	NO	10 093 219	10 093 578	Prupe.6G088300.1	AT1G58390	Hypothetical protein	-	1.3E-12	1	C:integral component of membrane
PAV04_REGINAg0203721	NO	10 095 690	10 096 082	Prupe.4G168300.1	AT5G29613	Cyclin-related protein		8.0E-82		
PAV04_REGINAg0203731	YES	10 097 190	10 099 049	Prupe.1G235900.1	AT1G03390	BAHD acyltransferase At5g47980-like		0.0E0	1	F:transferase activity, transferring acyl groups other than amino-acyl groups

PAV04_REGINAg0203741	YES	10 099 778	10 101 155	Prupe.7G054600.1	AT1G19220	Vinorine synthase-like		0.0E0	2	F:vinorine synthase activity; P:indole biosynthetic process
PAV04_REGINAg0203751	YES	10 102 625	10 104 375	Prupe.1G235900.1	AT3G44250	BAHD acyltransferase At5g47980-like		0.0E0	3	P:obsolete acyl-carrier-protein biosynthetic process; F:salutaridinol 7-O-acetyltransferase activity; P:alkaloid biosynthetic process
PAV04_REGINAg0203761	YES	10 108 430	10 109 727	Prupe.4G142000.1	AT1G24430	Vinorine synthase-like		0.0E0	1	F:transferase activity, transferring acyl groups other than amino-acyl groups
PAV04_REGINAg0203771	YES	10 115 267	10 116 971	Prupe.2G083900.1	AT1G31490	Vinorine synthase-like		1.8E-62	1	F:transferase activity, transferring acyl groups other than amino-acyl groups
PAV04_REGINAg0203791	YES	10 121 769	10 124 356	Prupe.4G140900.1	AT3G12960	Hypothetical protein	Unknown	1.2E-35	2	F:phosphatidylcholine binding; F:disaccharide binding
PAV04_REGINAg0203801	NO	10 125 158	10 125 582	Prupe.2G178200.1	AT4G21770	Hypothetical protein	-	2.3E-28		
PAV04_REGINAg0203811	NO	10 128 288	10 129 135	Prupe.7G129900.1	AT5G38130	BAHD acyltransferase At5g47980-like		0.0E0	1	F:transferase activity, transferring acyl groups other than amino-acyl groups
PAV04_REGINAg0203821	NO	10 129 184	10 129 387	Prupe.2G010800.1	AT2G19710	BAHD acyltransferase At5g47980-like		7.0E-32	1	F:transferase activity, transferring acyl groups other than amino-acyl groups
PAV04_REGINAg0203831	YES	10 131 063	10 131 627	Prupe.2G229700.1	AT3G42640	Transcription factor interactor and regulator CCHC(Zn) family		8.9E-127	2	F:nucleic acid binding; F:zinc ion binding
PAV04_REGINAg0203841	YES	10 131 629	10 135 406	Prupe.4G141500.1	AT4G00690	BAHD acyltransferase At5g47980-like		4.9E-32	3	P:obsolete acyl-carrier-protein biosynthetic process; F:salutaridinol 7-O-acetyltransferase activity; P:alkaloid biosynthetic process
PAV04_REGINAg0203851	YES	10 143 403	10 143 693	Prupe.4G140900.1	AT3G12960	Hypothetical protein	Unknown	8.7E-66	2	F:phosphatidylcholine binding; F:disaccharide binding
PAV04_REGINAg0203861	YES	10 144 043	10 150 300	Prupe.4G141000.1	AT5G26360	T-complex protein 1 (TCP-1) subunit gamma		0.0E0	4	C:chaperonin-containing T-complex; F:ATP binding; F:unfolded protein binding; P:protein folding
PAV04_REGINAg0203871	NO	10 158 700	10 159 093	Prupe.4G141100.1	AT2G24810	Thaumatin		1.9E-46	2	C:extracellular region; P:defense response

(1) Based on transcriptomic analyses, column to know if the gene is expressed in 'Regina' and 'Garnet', the parental cultivars of our plant material .

(2) Physical position (start and end) of the gene on the 'Regina' genome (in bp)

The candidate genes highlighted in grey are within the 68 kb-interval obtained with the fine mapping of the QTL.
